# Targeting Multilayered Metabolic Networks in Brain Diseases: Emerging Perspectives on Nanodelivery Strategies

**DOI:** 10.1002/advs.202503645

**Published:** 2025-09-25

**Authors:** Jingyi Zhou, Chen Jiang

**Affiliations:** ^1^ Department of Pharmaceutics School of Pharmaceutical Sciences Fudan University Key Laboratory of Smart Drug Delivery Ministry of Education State Key Laboratory of Brain Function and Disorders MOE Frontiers Center for Brain Science Shanghai 201203 China

**Keywords:** brain diseases, metabolism regulation, multilayered metabolic networks, nanodelivery strategies

## Abstract

Brain metabolism is uniquely regulated, and alterations in its metabolic networks often serve as critical drivers of the onset and progression of brain diseases. Therapeutic strategies that target these metabolic changes are regarded as fundamental to disease intervention. In complex metabolic networks, multi‐level metabolic dysregulation typically initiates a shared pathological process: the disruption of core cell metabolism leads to impaired cell–cell interactions, ultimately promoting the development of a malignant microenvironment that supports disease progression. This process encompasses complex mechanisms such as substance transport, cell signaling, and the dynamic regulation of the microenvironment. Smart nanodelivery systems, with their versatility, responsiveness, and modularity, can precisely modulate these dynamic metabolic networks in brain diseases, guided by the underlying pathological mechanisms. In this review, the metabolic network characteristics associated with brain diseases is summarized and the use of nanodelivery systems and their combinations are explored for metabolic regulation, aiming to establish a novel therapeutic paradigm.

## Graphical abstract

Brain diseases involve multilayered metabolic disruptions that reshape cellular interactions and microenvironments. This review outlines core metabolic features across disease states and presents emerging nanodelivery strategies as precision tools to reprogram pathological metabolism. By bridging metabolic logic and smart nanomedicine, it offers a systems‐level framework for targeted therapeutic intervention in the brain.

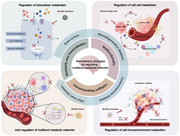

## 1 Introduction

Although the brain accounts for only 2% of total body weight, it consumes approximately 20%–25% of the body's oxygen at rest, reflecting its exceptionally high metabolic demand.^[^ ^1^ ^]^ This vigorous metabolic activity is sustained by complex structural compartmentalization and diverse cellular populations, collectively forming a dynamic and finely tuned metabolic network. As a central regulatory interface, the blood–brain barrier (BBB) maintains cerebral metabolic homeostasis by selectively transporting essential substrates such as glucose, lipids, and amino acids.^[^ ^2^ ^]^ Once within the brain parenchyma, these substrates are metabolized by various cell types—particularly neurons, astrocytes, and microglia—through distinct intracellular pathways and intercellular coupling mechanisms, thereby supporting systemic metabolic stability.

Under pathological conditions, this metabolic network becomes disrupted at multiple levels. Intracellular metabolic imbalance, impaired energy exchange between cells, and perturbations in the metabolic microenvironment act synergistically to initiate a “pathological metabolic network” centered on metabolic reprogramming. This remodeled network drives the sustained progression of brain diseases. For instance, neurodegenerative diseases and brain tumors often exhibit a similar pathological pattern: aberrant glucose metabolism in key cells impairs the metabolic functionality of neighboring populations, reshaping the microenvironment and exacerbating disease progression through positive feedback loops.

Abnormal metabolism thus represents not only a hallmark of brain disorders but also a central driving force behind their development, making it an increasingly important target for diagnosis and therapy. Clinically, positron emission tomography (PET) using fluorine‐18‐labeled fluorodeoxyglucose (18F‐FDG) has become a widely adopted technique for early diagnosis and lesion localization in various brain diseases, as it provides a direct assessment of glucose metabolic abnormalities .^[^ ^3^, ^4^, ^5^ ^]^ Despite advances in small‐molecule drugs aimed at modulating brain metabolism, several major obstacles remain. First, the BBB poses a significant barrier to the efficient delivery and accumulation of therapeutic agents at the lesion site, limiting drug efficacy. Second, the intricate and region‐specific intercellular networks in the brain are difficult to precisely modulate using conventional pharmacological approaches, which may instead trigger widespread metabolic disruption and compromise vital physiological functions. Moreover, the metabolic pathways of the brain are characterized by high degrees of coupling and dynamic compensation, making it difficult for single‐target interventions to align with the underlying pathological complexity, often resulting in limited clinical benefit.

In this context, smart nano‐delivery systems offer promising solutions. Through rational design, nanocarriers can significantly enhance drug penetration across the BBB and enable cell‐specific targeting, thereby achieving multi‐level and multi‐process metabolic intervention. Such systems can co‐deliver multiple therapeutic agents to simultaneously modulate intracellular metabolism, intercellular metabolic interactions, and the surrounding microenvironment, achieving coordinated multi‐target regulation. Some functional nanomaterials even possess intrinsic therapeutic activities, and their capacity for spatiotemporal control over drug release allows for adaptive responses to the evolving pathological metabolic landscape.

**Scheme 1:**
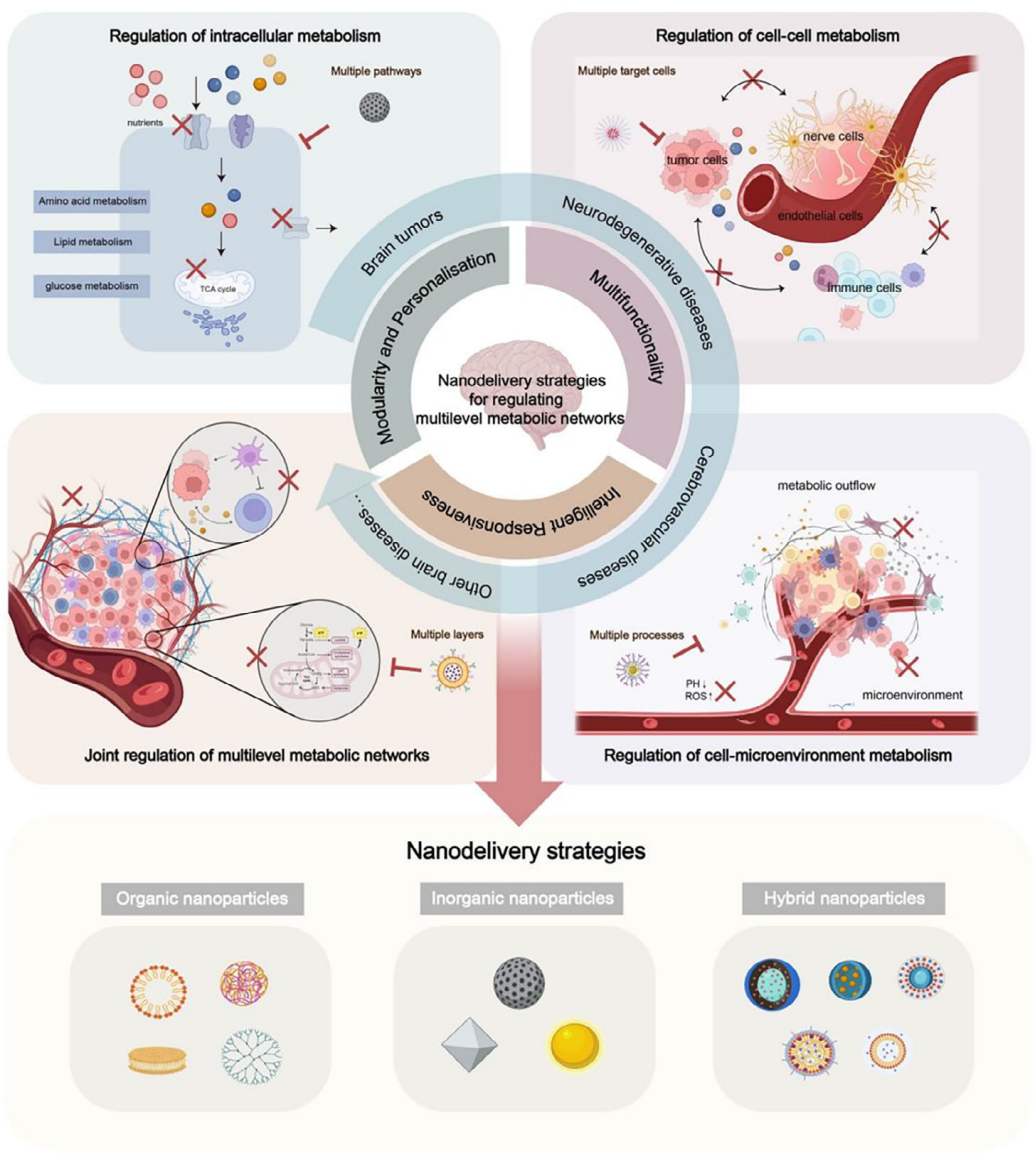
Nanodelivery strategies for regulating multilevel metabolic networks.

Based on these considerations, this review systematically summarizes recent advances in nano‐delivery strategies for regulating brain metabolism, focusing on their potential for multi‐target delivery and complex network interventions, while integrating the characteristic metabolic features of brain diseases (**Scheme** **1**). This work provides valuable theoretical and practical guidance for the development of nano‐enabled diagnostic and therapeutic approaches for brain disorders.

## 2 Abnormal Metabolic Networks and Brain Diseases

Metabolic processes extend beyond the intracellular environment and are finely regulated through cell‐cell and cell‐microenvironment metabolic networks. Nutrients are initially transported to neurons and glial cells via brain endothelial cells to meet the energy demands of the brain. Different types of brain cells operate interdependently, coordinating the transport, utilization, and clearance of substances within the brain. Simultaneously, cellular metabolites play a key role in maintaining microenvironmental homeostasis, directly influencing cell fate and the metabolic balance.

At disease onset, pathological changes often impair cellular metabolism, leading to dysfunction across multicellular populations and the collapse of the metabolic microenvironment, accelerating the progression of various neurological disorders. Therefore, in this section, we focus on the metabolic mechanisms underlying major neurological disorders, emphasizing their unique characteristics in metabolic regulation. In‐depth analysis of the dysregulation of metabolic networks in these pathological states not only reveals the underlying molecular mechanisms but also provides new ideas for personalized therapy.

### 2.1 Abnormal Intracellular Metabolism

Intracellular metabolism refers to the conversion of nutrients into energy and the biosynthesis of molecules essential for cellular function and stability through enzymatic reactions. It encompasses both energy metabolism and anabolic metabolism, which together provide cells with ATP and essential metabolites. Dysregulation of intracellular metabolic pathways, particularly those involving glucose, lipid, and amino acid metabolism, is closely associated with the onset of brain diseases.

Metabolic reprogramming plays a pivotal role in the pathogenesis of various brain diseases, particularly in brain tumors.^[^ ^6^ ^]^ Tumor cells exhibit significantly increased metabolic activity to meet the high energy demands required for their rapid proliferation and metastasis. For example, glioma cells display the “Warburg effect,” preferentially generating energy through aerobic glycolysis instead of mitochondrial oxidative phosphorylation (OXPHOS), even when sufficient oxygen is available.^[^ ^7^ ^]^ This metabolic pattern facilitates tumor cell proliferation in the short term; however, the accumulation of lactic acid (LA) acidifies the intracellular environment,^[^ ^8^ ^]^ disrupting pH homeostasis, which, in turn, enhances metabolic adaptation and treatment resistance in tumor cells. Additionally, the regulation of lipid metabolism in glioma cells is critical. By modulating fatty acid synthesis and metabolism,^[^ ^9^ ^]^ tumor cells support rapid membrane turnover and continuously active signaling, which promotes their proliferation, survival, and malignant progression. Moreover, the conversion of glutamine to glutamate facilitates glutathione (GSH) synthesis, aiding tumor cells in mitigating oxidative stress and enhancing survival.^[^ ^10^ ^]^

Certain key cells undergo progressive metabolic loss of function under pathological conditions as a way to drive disease processes, such as neurodegenerative diseases. In Alzheimer's disease (AD), endothelial metabolic dysfunction significantly reduces glucose supply, impairing neuronal energy metabolism, particularly in regions critical for cognitive function, such as the hippocampus and frontal cortex.^[^ ^11^ ^]^ Disturbed glucose–lipid interactions promote β‐amyloid plaque deposition and tau hyperphosphorylation, further accelerating the development of neurodegenerative lesions and intensifying pathological processes.^[^ ^12^ ^]^ In Parkinson's disease (PD), mitochondrial dysfunction and elevated oxidative stress similarly disrupt neuronal metabolism,^[^ ^13^ ^]^ reducing ATP production and impairing energy balance and metabolic homeostasis. The overactivation of oxidative stress not only exacerbates metabolic imbalance but also facilitates the aggregation of α‐synuclein, culminating in neuronal apoptosis and accelerating disease progression. Excitotoxicity, driven by abnormal glutamate metabolism, is common in both diseases, with NMDA receptor overactivation contributing to neuronal injury. Chronic imbalances in amino acid metabolism, particularly glutamate metabolism,^[^ ^14^ ^]^ exacerbate excitatory neurotoxicity and then drive neurodegeneration.

Unlike such chronic functional deficits, brain cells may experience acute metabolic collapse in response to sudden pathological changes, resulting in a significant reduction in ATP synthesis and rapid alterations in their metabolic functions. Ischemic stroke (IS) is a typical cerebrovascular condition characterized by this phenomenon. Disruption of glucose and oxygen supply impairs cellular energy production pathways, disrupting metabolic homeostasis and leading to ionic imbalance, membrane damage, and the activation of oxidative stress.^[^ ^15^ ^]^ This metabolic imbalance further exacerbates neuronal and glial cell dysfunction, causing extensive brain tissue damage and cell death. Following ischemia, intracellular metabolic functions are severely restricted, and the lack of sufficient energy hampers the initiation of effective brain cell repair mechanisms.

Several abnormal cellular metabolic states are strongly linked to brain disorders, including functional and seizure‐related disorders such as depression, anxiety, and epilepsy. In functional disorders like depression and anxiety, cerebral glucose metabolism is markedly reduced, particularly in the prefrontal cortex, a crucial region for emotion regulation. This metabolic deficiency directly affects the synthesis and release of neurotransmitters, leading to disturbances in emotional regulation, which subsequently contribute to functional psychiatric disorders.^[^ ^16^ ^]^ In seizure disorders such as epilepsy,^[^ ^17^ ^]^ the energy demands of the focal area are significantly elevated. During acute seizures, the rapid depletion of glucose in the brain causes a local energy deficit, which further increases neuronal excitability and lowers the depolarization threshold, thereby triggering excessive neuronal discharges and promoting the onset and persistence of seizures.

Altogether, diverse intracellular metabolic abnormalities disrupt energy supply and biosynthetic processes, thereby driving the pathological changes underlying brain diseases. Comprehensive investigation of these metabolic mechanisms offers critical insights into the pathological processes of brain disorders and offers new avenues for early diagnosis and targeted treatment.

### 2.2 Abnormal Cell–Cell Metabolic Interactions

Intracellular metabolic processes supply energy and essential substances to brain cells, while intercellular metabolic interactions coordinate the functions of different cell types, thereby maintaining brain tissue homeostasis. The diverse cellular environment of the brain—comprising neurons, glial cells, and brain endothelial cells—ensures normal brain function by exchanging substances and transferring metabolites. In disease states, metabolic imbalances between cells often accelerate disease progression by disrupting synergistic intercellular interactions and exacerbating pathological processes.

In glioblastoma (GBM), tumor cells domesticate other cellular components of the various microenvironments to “protect” tumor growth, infiltration, and immune escape. To meet their high nutrient demands, brain endothelial cells circumvent the physiological constraints of the BBB by upregulating key metabolite transporters, such as glucose transporters (GLUT1).^[^ ^18^ ^]^ Concurrently, glioma cells hijack neural circuits in the brain, diverting neural resources at the expense of cognitive functions, thereby promoting tumor growth.^[^ ^19^ ^]^ In addition, astrocytes enhance energy metabolism by delivering exogenous mitochondria to tumor cells.^[^ ^20^ ^]^ Studies have shown that metabolic interactions between tumor cells and local immune cells establish a complex immune‐metabolic feedback loop.^[^ ^21^ ^]^ In concrete terms, tumor cells reprogram immune cells to adopt a suppressive phenotype, creating a “cold” immune microenvironment conducive to glioma progression.^[^ ^22^ ^]^ Furthermore, certain immune cells serve as reservoirs of energy substrates for tumor cells, further supporting tumor survival and proliferation. Notably, the inability of tumor cells to access exogenous cholesterol due to the limitations of the BBB is considered a potential therapeutic vulnerability.^[^ ^23^ ^]^ Macrophages have been shown to supply cholesterol to tumor cells,^[^ ^24^ ^]^ a phenomenon that is relatively rare in peripheral tumors, highlighting the unique mechanisms regulating nutrient uptake and energy metabolism in glioblastoma.

The pathological features of chronic neurodegenerative diseases and acute cerebrovascular diseases are markedly different; however, they both share commonalities in certain intercellular interactions and involve a vicious cycle of interactions triggered by degeneration of the metabolic function of each cell. Both diseases show disturbances in endothelial‐neuronal interactions, either through sudden disruption of blood flow or gradual loss of function. In the early stages of a stroke, brain endothelial cells temporarily alleviate metabolic stress through compensatory mechanisms, such as upregulation of transporter proteins. However, prolonged ischemia ultimately leads to impaired nutrient transport, similar to that seen in neurodegenerative diseases,^[^ ^25^ ^]^ resulting in long‐term metabolic disturbances in neurons and contributing to functional deterioration. Astrocytes supply energy to neurons via lactate and amino acid metabolism, acting as guardians. However, with disease progression, astrocytic function declines, metabolic compensation is lost, and neuronal damage worsens due to the ongoing inflammatory response.^[^ ^26^ ^]^ Similarly, microglia are also evident in both diseases. Constantly activated microglia release pro‐inflammatory factors and reactive oxygen species, amplifying neuronal damage and intensifying the chronic inflammatory response,^[^ ^27^ ^]^ thereby further accelerating neuronal damage and functional degeneration.^[^ ^28^, ^29^ ^]^

In functional disorders such as epilepsy, the metabolic regulation of brain endothelial cells also plays a critical role in disease progression.^[^ ^30^ ^]^ During epileptic seizures, the energy supply of the brain is disrupted and the endothelial cell transporter function is impaired, reducing glucose transport efficiency across the BBB. The overconsumption of glucose by neurons, coupled with the failure of glial cells to provide timely energy support, further exacerbates the persistence of seizures.^[^ ^31^ ^]^

In summary, during disease states, intracellular metabolic patterns become dysregulated, which disrupts energy supply and material exchange through intercellular metabolic interactions, establishing a pathological feedback loop. Formerly compensatory mechanisms that once maintained homeostasis are reprogrammed to drive pathological processes, causing feedback loops and cascade effects that accelerate the progression of diseases.

### 2.3 Abnormal Cell–Microenvironment Metabolic Interactions

Cell‐microenvironment metabolic activity refers to the metabolic signaling and reciprocal regulation between brain cells and their surrounding microenvironment, including the extracellular matrix, metabolic effluxes, and gaseous environment. This metabolic interplay not only sustains cell function and survival but also plays a critical role in preserving brain tissue homeostasis. In pathological states, alterations in the microenvironment disrupt this metabolic balance, impairing cellular function, driving disease progression, and contributing to the exacerbation of disease development.

For instance, in brain tumors, tumor cells cause microenvironment acidification by overproducing LA, which not only increases tumor cell invasiveness but also suppresses immune cell function and weakens the anti‐tumor immune response.^[^ ^32^ ^]^ Simultaneously, tumor cells regulate extracellular matrix remodeling by reprogramming glutamine and lipid metabolism, promoting neovascularization, and supplying essential nutrients and oxygen to tumor cells. These metabolic activities are interconnected, establishing a “fertile soil” that supports tumor growth. Cell‐microenvironment metabolic interactions not only drive tumor progression but also drive tumor adaptation through bi‐directional shaping, enhancing malignant properties and further complicating the disease.

Similarly, in neurodegenerative diseases, abnormalities in glial cell metabolism, particularly lactate and lipid metabolite accumulation, lead to local microenvironment acidification^[^ ^33^ ^]^ and suppress immune cell function. Additionally, this metabolic imbalance promotes the release of inflammatory mediators, culminating in a self‐perpetuating malignant metabolic environment.

Acute ischemia induces a highly hypoxic microenvironment, with further energy supply disruption leading to metabolite accumulation, exacerbating microenvironmental deterioration. Simultaneously, hypoxia promotes the release of pro‐inflammatory factors from cells, altering the local acidity and microvascular permeability, thereby exacerbating ischemic injury. This metabolic disruption also contributes to BBB breakdown and impairs brain tissue recovery. The interplay between metabolic imbalance and microenvironmental changes not only limits cellular energy supply but also leads to widespread neuronal death and tissue damage, driving disease progression.

Metabolic imbalances in the cellular microenvironment also play an important role in functional disorders, primarily in the dysregulation of the local environment. Affective regulation disorders, such as depression, primarily involve metabolic disturbances in the emotion‐related brain regions, particularly the prefrontal cortex. Reduced glucose metabolism impairs neuronal metabolic function, with the accumulation of metabolic waste products in the microenvironment further exacerbating local environmental deterioration.

It is evident that cell‐to‐cell metabolic signals are mediated by the microenvironment. Aberrant metabolic states initiate bidirectional feedback loops between cells and the microenvironment through dynamic metabolic networks, ultimately establishing a new “pathological homeostasis” that sustains and promotes disease progression. This complex and persistent pathological state makes the disease more resistant to single‐target regulation, thereby posing a significant challenge to treatment.

Therefore, addressing the complex metabolic networks involved in brain diseases requires multi‐targeted systemic regulatory strategies. By comprehensively modulating the function of multiple key factors in these networks, global regulation of the disease can be achieved, representing a potential breakthrough in the treatment of brain diseases.

## 3 Characteristics and Advantages of Nano‐Delivery Strategies

### 3.1 Multifunctionality

Engineered nanocarriers exhibit outstanding functional properties. Through structural modification, encapsulation, or adsorption, nanocarriers can optimize drug properties, enhance their ability to cross the BBB, and increase drug accumulation at focal sites. Furthermore, through specific surface modification and biomimetic encapsulation technologies, nano‐delivery systems can precisely regulate metabolic processes in target cells or achieve synchronized regulation of multiple cell types, thus enhancing therapeutic efficacy.

Importantly, some nanocarriers possess intrinsic therapeutic functions. For example, manganese dioxide (MnO_2_) nanoparticles catalyze the decomposition of excessive hydrogen peroxide in the tumor microenvironment (TME), alleviating hypoxia and inhibiting tumor metabolic adaptation. By leveraging their sensitive chemical structures or the catalytic properties of metals, nanocarriers can directly regulate metabolism while delivering drugs, thereby producing a synergistic therapeutic effect that surpasses the sum of individual contributions.

### 3.2 Intelligent Responsiveness

Responsive design endows nanocarriers with the ability to adapt to the dynamic metabolic microenvironment and modulate drug release accordingly. For instance, pH‐sensitive polymers such as poly(methacrylic acid‐co‐vinyl pyridine) (PMAA‐VP) and polypeptide‐based nanomaterials undergo structural changes in the acidic TME, triggering the release of encapsulated drugs. Thermo‐responsive carriers like poly(N‐isopropylacrylamide) (PNIPAM) adjust drug release rates in response to local temperature fluctuations. Enzyme‐responsive systems utilize matrix‐degrading enzymes, including matrix metalloproteinases (MMPs), as stimuli to selectively release therapeutic agents. Such mechanisms facilitate the precise regulation of spatial and temporal drug release patterns, tailored to the metabolic environment and the molecular characteristics of specific metabolic sites. Through advanced environmental sensing, these systems effectively mitigate the malignant properties of the local metabolic environment. By responding to changes in metabolic characteristics in different regions under pathological conditions, nanocarriers can implement sequential, intelligent drug release protocols and dynamically modulate the metabolic process, thereby optimizing therapeutic outcomes.

### 3.3 Modularity and Personalization

Modular design endows nanocarriers with flexible multi‐component integration capabilities, enabling them to provide personalized therapeutic regimens based on the characteristics of the metabolic networks. Given the complexity of metabolic networks, encompassing diverse mechanisms and targets associated with distinct effector cell types and drug action profiles, nanocarriers can effectively incorporate various therapeutic agents such as small molecules, genes, and antibodies, owing to their unique compatibility and integrative capacity. For example, multilayer core‐shell nanoparticles can separately encapsulate different drugs to achieve sequential release in both temporal and spatial dimensions. Detachable linkers can be utilized to co‐deliver small‐molecule drugs and nucleic acids within a single carrier for combination therapy. Furthermore, functional modifications can achieve targeted delivery to distinct cell types, thereby establishing a composite therapeutic platform capable of simultaneously modulating multiple key targets.

This nano‐delivery strategy demonstrates significant potential for metabolic regulation in complex brain diseases, such as neurodegenerative disorders and brain tumors. It enables precise targeting of multiple cell populations and intervention in various metabolic pathways simultaneously, enabling regulation across multiple metabolic levels. This approach is particularly well‐suited to the complexity and dynamic nature of metabolic networks in brain diseases.

Notably, such nanoplatforms are highly versatile and tunable, enabling the flexible adjustment of their components based on the specific requirements of different disease types, individual variations, and metabolic processes. This adaptability offers new possibilities for universal combination therapies, suggesting broader application scenarios and improved therapeutic outcomes.

## 4 Regulation of Brain Metabolic Networks for Nanodelivery Strategies

By leveraging the advantages of nano‐delivery systems, precise regulation of brain metabolic networks becomes feasible, enabling multi‐target intervention and multi‐component co‐delivery of diverse therapeutic agents. This approach facilitates the restoration of intracellular metabolic homeostasis, regulation of intercellular metabolic interactions, and modulation of microenvironmental characteristics, systematically correcting metabolic imbalances. Together, these mechanisms provide innovative therapeutic solutions for complex brain diseases. This section summarizes diverse nano‐delivery strategies (**Table** **1**) that are promising for the development of novel therapeutic approaches for brain diseases.

**Table 1:** Nanodelivery strategies based on brain metabolic network regulation.

Diseases	Metabolic intervention levels	NPs	Type of preparation	Research method	Administration method	Therapeutic strategy	References
GBM	Intracellular	GOx@MnCaP@fibrin	Fibrin gel	Subcutaneous U87 tumor surgical resection model	In situ spraying	Depletion of intracellular glucose	[35]
		MAG@EB	Bionic coated nanoparticles	Orthotopic C6 tumor model	Intravenous injection	Depletion of intracellular glucose	[36]
		ApoE‐MT/siPKM2 NC	Nanocapsules	Orthotopic U87 tumor model	Intravenous injection	Inhibition of glycolysis	[38]
		HM‐NPs@G	Bionic coated nanoparticles	Orthotopic U87 tumor model, Patient‐derived X01 xenograft model	Intravenous injection	Inhibition of mitochondrial function	[39]
		PAMSe	Nanomotors	Orthotopic GL261 tumor model	Intravenous injection	Inhibition of mitochondrial function	[41]
	Cell‐cell	Glycosylated A7R nanodisk	Nanodisk	Orthotopic U87 tumor model	Intravenous injection	Delivery via endothelial cell‐expressed GLUT1	[43]
		pOEI/DOX/ATP NPs	Nanoparticles	Orthotopic U87 tumor model	Intravenous injection	Delivery via endothelial cell‐expressed LAT1	[44]
		GGT‐activatable nanoprobe	Nanoprobe	Orthotopic U87, C6, G422 tumor model	Intravenous injection	Delivery via endothelial cell‐expressed GGT	[45]
		NPsiGLUT3	Nanoparticles	Orthotopic U87, U251 tumor model	Intravenous injection	Inhibition of endothelial cell‐tumor cell glucose transport	[47]
		sCND	Carbon nanodots	Orthotopic U87 tumor model	Intravenous injection	Inhibition of endothelial cell‐tumor cell glucose transport	[48]
		nRs	Hydrogel	Orthotopic GL261 tumor model and postsurgical recurrence model	In situ spraying	Inhibition of macrophage‐tumor cell cholesterol supply	[51]
	Cell‐microenvironment	M@HLPC	Bionic coated nanoparticles	Orthotopic U251 tumor model, Patient‐derived xenograft model	Intravenous injection	Inhibition of tumor cell lactate efflux	[53]
		Gel@B‐B	Thermogels	Orthotopic GL261 tumor model	In situ injection	Inhibition of tumor cell lactate efflux	[54]
		lactate‐loaded silica NPs	Nanoparticles	Orthotopic C6 tumor model	In situ injection	Lactate overload in the tumor microenvironment	[56]
		ACS NPs	Nanoparticles	Orthotopic GL261 tumor model	Intravenous injection	Inhibition of metabolite ADO production	[58]
	Multilevel	2‐DG/aV‐siCPT1C NC	Nanocapsules	Orthotopic U87 tumor model	Intravenous injection	Endothelial GLUT1‐mediated delivery blocks tumor glycolysis, fatty acid oxidation, and angiogenesis.	[61]
		BSA/LF NPs	Bionic nanoparticles	Orthotopic GL261 tumor model	Intravenous injection	Simultaneous inhibition of glycolysis and NADH‐ATP metabolism to regulate the immune microenvironment	[64]
		MBS‐Abz	Nanosilver	Orthotopic C6 tumor model	Intravenous injection	Simultaneous inhibition of glycolysis and mitochondrial function	[65]
AD	Intracellular	REn	Micelles	APP/PS1 model	Intravenous injection	Recovery of neuronal mitochondrial function and promotion of glucose metabolism	[67]
		PdH	Nanoparticles	3xTg‐AD model	Stereotaxic injection	Recovery of neuronal mitochondrial function	[69]
	Cell‐cell	PM	Nanoparticles	APP/PS1 model	Intravenous injection	Delivery via endothelial cell‐expressed GLUT1	[71]
		FTY@ Man NP	Oral nanoparticles	FAD^4T^, APP/PS1, Aβ‐injection model	Oral gavage	Delivery via endothelial cell‐expressed GLUT1	[72]
		Gal‐NP@ siRNA	Nanoparticles	APP/PS1 model	Intravenous injection	Delivery via endothelial cell‐expressed GLUT1	[73]
		VLC@ Cur‐NPs	Nanoparticles	APP/PS1 model	Intravenous injection	Modulation of pericyte function and restoration of pericyte‐neuron substance transport	[75]
		GAF NPs	Gold nanocages	APP/PS1 model	Intravenous injection	Rebalancing of glucose metabolism between microglia and neurons	[81]
	Cell‐microenvironment	APLB/CUR	Micelles	APPswe/PSEN1dE9 model	Intravenous injection	Elimination of microenvironmental ROS and remodeling of microglial function	[82]
	Multilevel	Nano‐Brake	Liposomes	5xFAD model	Intravenous injection	Simultaneous rescue of mitochondrial function in endothelial cells, neurons and microglia	[83]
		DA‐PPHATK@PDA	Peptide‐drug conjugate	APPswe/PSEN1dE9 model	Intravenous injection	Enhancement of microglial function, induction of astrocyte‐to‐neuron trans differentiation, and clearance of microenvironmental ROS	[84]
PD	Intracellular	RVG29@AHM@Pt/CeO_2_	Monoatomic Catalysts	Parkinson's disease model (MPTP)	Intravenous injection	Restoration of neuronal mitochondrial function and clearance of excess ROS	[68]
AS	Intracellular	MHT	Nanoparticles	MCAO model	Intravenous injection	Restoration of neuronal mitochondrial function and ROS clearance	[87]
		SPNPs	Hydrogels	MCAO model	Intranasal administration	Restoration of neuronal mitochondrial function and ROS clearance	[88]
		TPP@ (CeO_2_+ROF)	Nano‐enzymes	MCAO model	Intravenous injection	Restoration of neuronal mitochondrial function	[92]
		NTUPs‐tPA	Bionic coated nanoparticles	Acute thromboembolic stroke model	Intravenous injection	Promotion of neuronal ATP and NADPH production	[93]
	Cell‐cell	PC NPs	Needle‐like nanostructures	BCCAO model	Intravenous injection	Delivery via endothelial cell‐expressed GLUT1	[94]
	Cell‐microenvironment	MFP	Bionic coated nanoparticles	tMCAO model	Intravenous injection	Increase of oxygen supply in the microenvironment	[96]
		Pt@LF	Bionic nanomotors	tMCAO model	Intravenous injection	Scavenging of ROS, iron chelation, and removal of excess NH4^+^ from the microenvironment	[99]
		S‐DCM‐NIR T‐(835)	Fluorescent nanoprobes	Acute thromboembolic stroke model	Intravenous injection	Response to cysteine luminescence in the microenvironment	[100]
	Multilevel	CPTK@ PMH	Bionic nano‐erythrocytes	tMCAO model	Intravenous injection	Inhibition of microglial polarization, restoration of neuronal glucose metabolism, and clearance of microenvironmental ROS	[101]

### 4.1 Brain Tumors

#### 4.1.1 Regulation of Intracellular Metabolism

In cancerous glial cells, heightened metabolic activity underpins sustained tumor growth. By disrupting such metabolic processes within tumor cells, metabolic starvation can be induced to suppress the overactive cellular metabolic state, thereby diminishing the growth drive of tumors.

Depleting existing nutrients within tumor cells can directly sever the energy supply, thus reducing their metabolic activity. Co‐delivering energy metabolism or pathway‐modulating drugs can further enhance their inhibitory effect on tumors. Employing enzymes to deplete metabolic substrates within tumor cells represents a promising strategy. For example, glucose oxidase (GOx) converts glucose or lactate into gluconic acid or pyruvate in the presence of oxygen while generating toxic hydrogen peroxide (H_2_O_2_).^[^ ^34^ ^]^ This process not only depletes metabolic substrates in tumor cells but also enhances the local acidic environment through acidification and reactive oxygen species (ROS) generation, offering a theoretical basis for designing multimodal therapeutic strategies.

However, enzymes are large macromolecular proteins susceptible to in vivo degradation, encountering significant challenges in crossing biological barriers. In situ biomimetic mineralization strategies facilitate the spontaneous formation of nanoparticles within the local TME, effectively preventing the premature degradation or inactivation of drugs and enzymes, a common issue in conventional methods. This approach offers precise control over the mineralization process, enabling modulation of the size, morphology, and surface properties of the nanoparticles, which in turn enhances their targeting efficiency, biocompatibility, and accumulation in tumor tissues. In this regard, Li et al.^[^ ^35^ ^]^ utilized in situ biomimetic mineralization to generate GOx‐loaded nanoparticles (GOx@MnCaP) by doping manganese into calcium phosphate, followed by incorporation into fibrin gel. After glioma surgery, the gel releases GOx at the tumor site, where it consumes glucose and generates H_2_O_2_, thereby inducing tumor cell death through a Mn^2+^‐mediated Fenton reaction. Moreover, biomimetic systems have been designed to maintain the metabolic activity of enzymes within the delivery system. For instance, self‐assembling nanoparticles encapsulating GOx, Mn^2+^, and arsenate, wrapped in exosomes derived from brain metastatic breast cancer (EB), significantly enhanced their ability to cross the BBB and improved enzyme stability.^[^ ^36^ ^]^

After entering the cell, metabolic substrates undergo transformation and utilization through a series of precisely regulated metabolic pathways. Multiple enzymes serve as core regulatory factors in this process, maintaining the stability and efficiency of the cellular metabolic networks. Disrupting or inhibiting the function of these key enzymes can effectively impair the normal flow of metabolic pathways.

**Figure 1:**
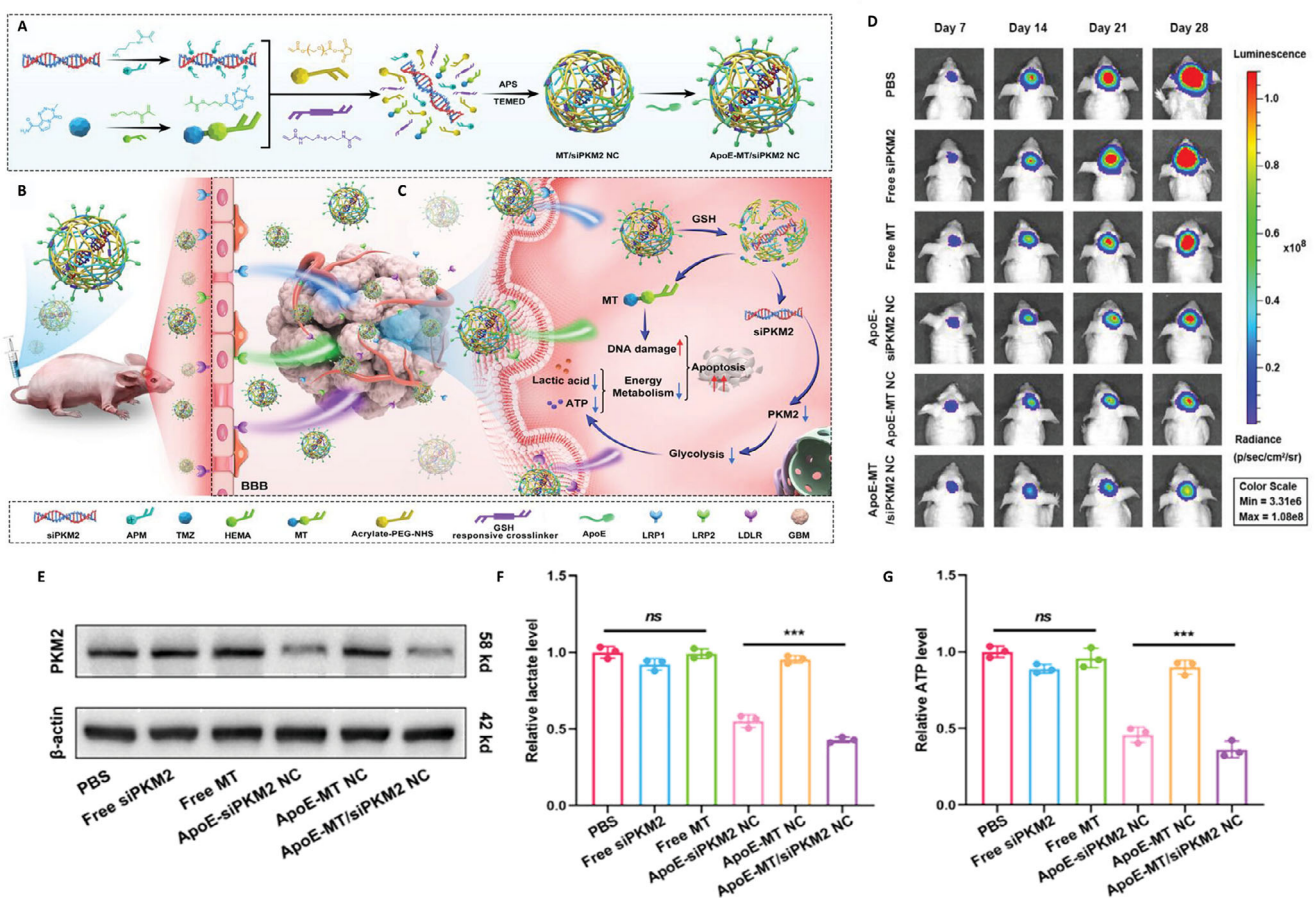
Dual‐targeted novel temozolomide nanocapsules encapsulating siPKM2 (ApoE‐MT/siPKM2 NC). A) Preparation process of ApoE‐MT/siPKM2 NC. B) The targeting process of ApoE‐MT/siPKM2 NC across the blood‐brain barrier (BBB). C) Intracellular release process of ApoE‐MT/siPKM2 NC. D) Biofluorescence imaging of orthotopic glioblastoma‐bearing mice following various treatments. E) Western blot assays to analyze the pyruvate kinase M2 (PKM2) protein expression level in U87 cells with different treatments. F,G) Statistical analysis of F) lactic acid and G) ATP levels after incubation with ApoE‐MT/siPKM2 NC.^[^ ^38^ ^]^ Copyright 2024, Wiley‐VCH.

In recent years, small‐molecule metabolic inhibitors have been reported to successfully disrupt the energy supply of tumor cells by inhibiting the activity of essential enzymes. However, the BBB presents a significant challenge for the direct delivery of these drugs to brain tumors. Nanomedicine systems, which enhance brain targeting and tumor accumulation, have emerged as a crucial strategy to overcome this obstacle. 3‐Bromopyruvate (3BP), an inhibitor of hexokinase (HK)‐II and glyceraldehyde‐3‐phosphate dehydrogenase (GAPDH), inhibits glycolysis and mitochondrial respiration, rapidly depleting ATP. Deng et al.^[^ ^37^ ^]^ developed a prodrug system based on α‐cyclodextrin (CD) to co‐deliver 3BP and the photosensitizer chlorin e6 (Ce6), combining photodynamic therapy with metabolic inhibition. In addition to conventional inhibitors, nanomedicine systems designed for siRNA delivery offer an effective strategy for targeting challenging proteins. Zhang et al.^[^ ^38^ ^]^ designed and synthesized a novel nanocapsule (ApoE‐MT/siPKM2 NC) to co‐deliver pyruvate kinase M2 siRNA (siPKM2) and temozolomide (TMZ), using siPKM2 as the core and a methyl methacrylate‐TMZ (MT) outer layer. The developed platform achieved dual targeting of the BBB and GBM through ApoE modification. Furthermore, the disulfide (DSF)‐bond cross‐linked GSH‐responsive linkage structure within the capsule enabled selective cleavage in the high GSH environment of glioma cells, ensuring precise drug release (**Figure** **1**).

Given the critical role of mitochondria in cellular energy metabolism, studies have increasingly focused on the development of nanomedicine systems that specifically target distinct metabolic sites, aiming to broadly intervene in and disrupt tumor metabolic processes. Zou et al.^[^ ^39^ ^]^ engineered a biomimetic nanomedicine (HM‐NPs@G) by encapsulating nanoparticles loaded with the OXPHOS inhibitor Gboxin within a hybrid membrane composed of cancer cells and mitochondria. This membrane enables dual targeting of both tumors and mitochondria via “self‐labeling” proteins and surface adhesion molecules. Additionally, considering that mitochondria produce approximately 90% of intracellular ROS, ROS‐responsive elements were incorporated to address the poor stability, short half‐life, and limited permeability of Gboxin,^[^ ^40^ ^]^ thus enabling rapid and precise drug release. Chen et al.^[^ ^41^ ^]^ designed a nanomotor based on a polycationic polymer with L‐arginine derivatives as monomers, driven by nitric oxide (NO). Within the GBM microenvironment, the high‐density guanidine groups in the nanomotor react with ROS/iNOS to produce NO. The elevated NO concentrations serve as a chemical attractant, guiding the nanomotor to exhibit chemotaxis toward the TME through its specific affinity for the enzyme substrate. By modifying the nanomotor with angiotensin‐converting enzyme‐2 (Ang) to specifically target brain endothelial cells and GBM cells and employing the targeting capability of triphenylphosphine (TPP) to direct the drug lonidamine (LND) to mitochondria, a stepwise targeting process was achieved: from brain endothelial cells to tumor cells to mitochondria, ultimately disrupting abnormal metabolic pathways.

#### 4.1.2 Regulation of Cell–Cell Metabolism

Brain endothelial cells are critical for maintaining brain function, serving as the “regulatory hub” for nutrient supply in the brain.^[^ ^42^ ^]^ During disease progression, they may undergo functional changes; concurrently, they can regulate transport efficiency and regulatory functions based on the metabolic status and needs of cells in different diseases, thereby influencing the metabolic balance of other cells through substrate supply.

The high energy demand of tumors drives endothelial cells to accelerate nutrient transport. This significant upregulation of metabolic substrate transporter proteins on the cell surface not only enhances the supply of metabolic substrates to the tumor but also presents an opportunity for targeted drug delivery. Strategies employing nutrient analogues as targeting ligands and the endothelial‐tumor cell interaction pathway for drug delivery have become widely employed. For example, Wang et al.^[^ ^43^ ^]^ designed a glycosylated A7R peptide derivative to enable peptide molecules to cross the BBB and effectively enter brain tissue by exploiting the GLUT1‐mediated glucose transport mechanism, which is highly expressed on endothelial cells. Meanwhile, the A7R peptide recognized the overexpressed vascular endothelial growth factor receptor 2 (VEGFR2) and neuropilin‐1 (NRP‐1) in the brain to achieve cascade‐targeting functionality. An et al.^[^ ^44^ ^]^ focused on the large amino acid transporter protein 1 (LAT1), which is significantly overexpressed in both BBB and glioma cells, and synthesized a high‐affinity substrate derivative (3CDIT), enabling the targeted delivery of various therapeutic agents. Interestingly, Li et al.^[^ ^45^ ^]^ directly exploited the metabolic characteristics of endothelial cells to achieve responsive transformation of the nanoprobe. γ‐Glutamyl transpeptidase (GGT), a key enzyme in amino acid metabolism, is significantly overexpressed in BBB‐associated cells.^[^ ^46^ ^]^ By modifying the γ‐glutamyl group on the surface of the neutral nanoprobe to enable cleavage by GGT, which in turn generates a positively charged primary amine group, the nanoprobe efficiently crosses the BBB via the adsorption‐mediated transcytosis‐phagocytosis pathway, avoiding the cytotoxicity associated with traditional cationic nanocarriers.

Another strategy involves disrupting the endothelial‐tumor cell interaction pathway by blocking the transporter function of endothelial cells, thereby limiting the energy supply of the tumor. This can be achieved by administering inhibitors or by downregulating the expression of transporter proteins using siRNA. Nano‐delivery systems not only enhance the targeting and bioavailability of small molecules but also protect siRNAs and improve their targeted silencing efficacy. For instance, Xu et al.^[^ ^47^ ^]^ employed an ionic lipid‐assisted PEG‐PLA nanoparticle system to effectively deliver specific GLUT3 siRNA to the tumor site, significantly reducing the metabolism and proliferative capacity of tumor cells. Furthermore, Wang et al.^[^ ^48^ ^]^ synthesized a small sugar‐derived carbon nanodot (sCND), which is highly water‐dispersible with functionalized residues on its surface. This material can cross the BBB and specifically target tumors via GLUT1. Since sCND cannot be phosphorylated to participate in glycolysis, it can competitively inhibit glucose uptake, thus limiting the energy supply of the tumor.

The metabolic status of immune cells in the brain, primarily microglia and macrophages, is influenced by the brain microenvironment and metabolic abnormalities in other cells, as well as by their role in maintaining brain homeostasis through the regulation of nutrient supply and metabolite feedback. Additionally, as immune effector cells, they are distinctive in their ability to influence the metabolic balance of other cells by secreting inflammatory factors and mediating immune responses, thereby contributing to the deterioration of the metabolic microenvironment.

**Figure 2:**
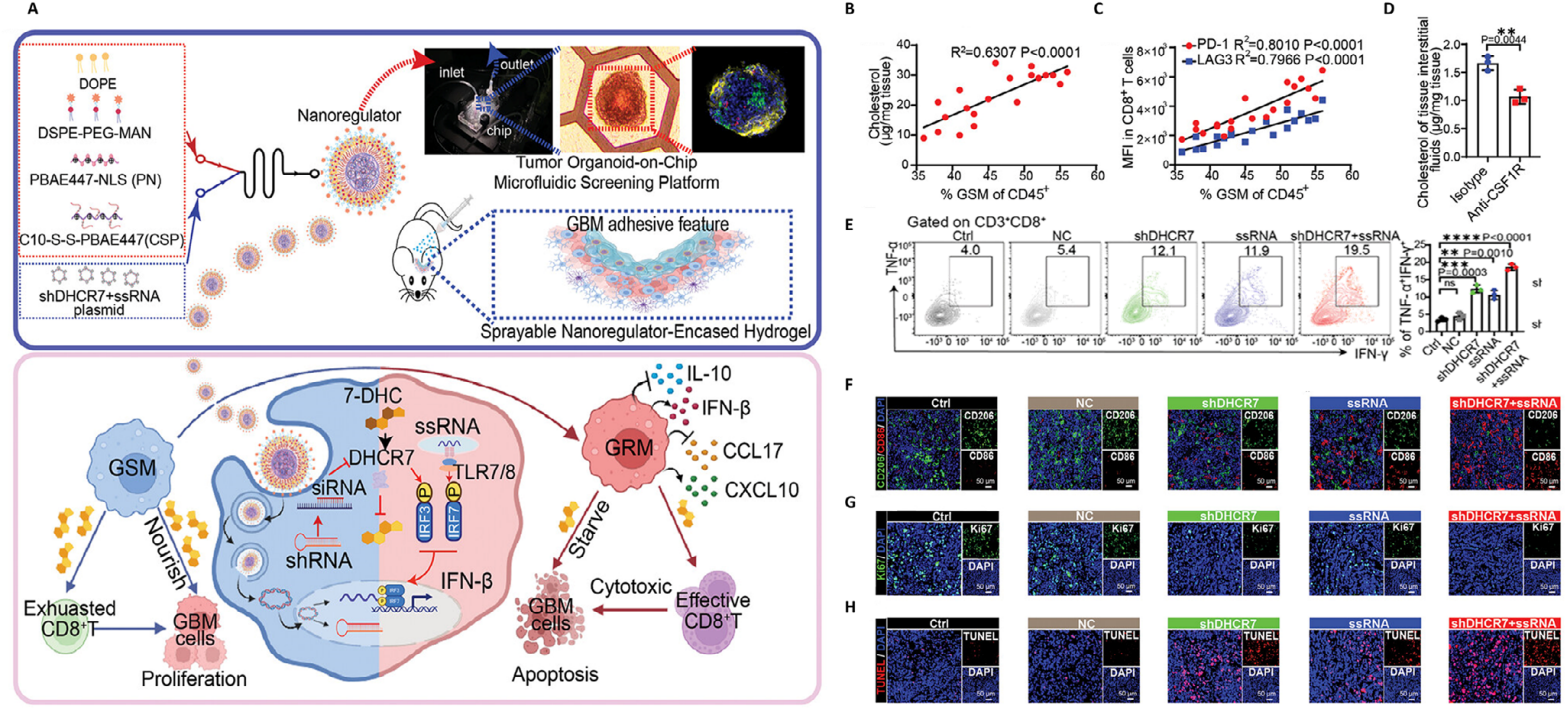
Intracavitary spraying of nanoregulator‐encased hydrogel modulates cholesterol metabolism of glioma‐supportive macrophage (GSMs) (shDHCR7+ssRNA@NRs). A) Schematic representation of shDHCR7^+^ssRNA@NRs used to regulate cholesterol metabolism in GSMs. B) Correlation analysis between cholesterol level and GSMs proportion in tumors. C) Correlation analysis between PD‐1/LAG3 expression and GSMs proportion. D) The cholesterol level in tumor tissue interstitial fluid. E) Flow cytometry analysis of the proportion of IFN‐γ^+^TNF‐α^+^ T cells. F) Immunofluorescence images of GSMs in different treatment groups. CD86, red; CD206, green; DAPI, blue. Scale bar, 50 µm. G) Ki67 and H) TUNEL staining of brain tissue sections.^[^ ^51^ ^]^ Copyright 2023, Wiley‐VCH.

In the glioma microenvironment, microglia and macrophages, collectively referred to as glioma‐supportive macrophages (GSMs), collaborate to influence tumor progression and immune evasion through complex metabolic interactions. The metabolic state of GSMs is regulated by metabolites secreted by tumor cells (e.g., LA, ATP, etc.) and by providing essential nutrients to support tumor cell growth. These metabolic interactions shape the immunosuppressive microenvironment of glioblastoma and create favorable conditions for tumor cell proliferation. Studies have demonstrated that cholesterol levels are significantly elevated in GBM tissues, with GSMs acting as a “cholesterol factory” to support tumor growth by enhancing cholesterol synthesis and efflux.^[^ ^49^ ^]^ In this process, GSMs not only supply cholesterol to the tumor but also suppress the immune response by promoting the depletion of CD8^+^ T‐cells, thereby facilitating immune evasion by the tumor.^[^ ^50^ ^]^ Based on this mechanism, a hydrogel system was developed^[^ ^51^ ^]^ encapsulating nanomodulators (NR) in an intracavity spray to precisely intervene in cholesterol metabolism in GSM and inhibit GBM growth. This strategy targets 7‐dehydrocholesterol reductase (DHCR7) in GSM using a degradable NR carrier to inhibit cholesterol biosynthesis and reduce the cholesterol supply from GSM to tumor cells, thereby limiting tumor cell survival and proliferation (**Figure** **2**).

#### 4.1.3 Regulation of Cell–Microenvironment Metabolism

Advances in tumor metabolism have revealed that the metabolites produced during cellular metabolism are not merely “waste.” These byproducts can be released into the microenvironment, where they participate in substance transport and metabolic regulation and even act as signaling molecules to regulate the functions and behaviors of surrounding cells.^[^ ^52^ ^]^ Consequently, targeting the regulation of the microenvironment offers a broad approach to addressing metabolically abnormal cell populations and provides new therapeutic strategies. For example, tumor cells competitively consume glucose and secrete LA, produced through glycolysis, into the microenvironment via transporters. The accumulation of LA in the microenvironment promotes macrophage polarization to an anti‐inflammatory phenotype and sustains the immunosuppressive function of Tregs, facilitating immune evasion. Additionally, cancer cells in hypoxic regions can uptake lactate via MCT1 and oxidatively metabolize LA to generate energy, creating a “lactate shuttle” phenomenon.

**Figure 3:**
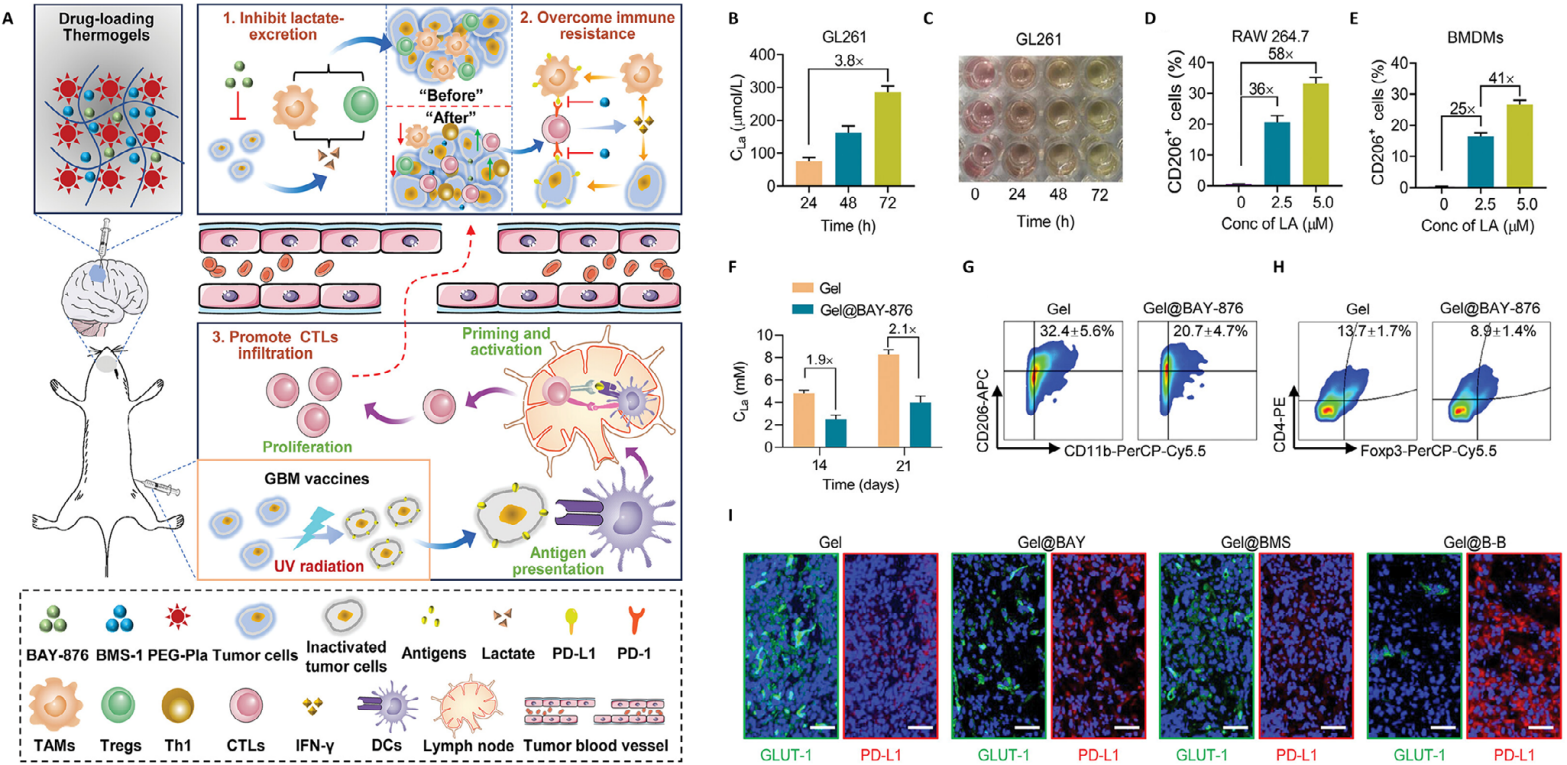
Metabolism/Immunity dual‐regulation thermogels. Schematic illustration of drug‐loading thermogels in alleviating immunosuppressive tumor microenvironment by inhibiting lactate excretion and overcoming immune resistance. B) Lactate concentration in medium of GL261 cells at different culture time. C) Image of GL261‐cultured plate. D,E) Flow cytometry analysis of M2 macrophages in lactate‐treated RAW264.7 cells and bone marrow‐derived macrophages (BMDMs). F) Lactate concentration in tumor tissue of mice at 14 and 21 d. G,H) Flow cytometry analysis of M2 macrophages and regulatory T cells (Tregs). L) immunofluorescence section of tumor tissues stained with DAPI (blue), PD‐L1 (red), and GLUT1 (green).^[^ ^54^ ^]^ Copyright 2024, The Authors. Advanced Science published by Wiley‐VCH.

Consequently, the inhibition of lactate production, exocytosis, and internalization disrupts the metabolic symbiosis, leading to the blockage of tumor proliferation and the induction of tumor cell apoptosis. Lu et al.^[^ ^53^ ^]^ revealed significantly elevated lactate levels in the in vivo microenvironment of glioma patients and tumor‐bearing mice and designed a biomimetic nano‐delivery system using lactate as an energy substrate. The system was loaded with lactate oxidase (LOX) via the extracellular envelope of glioma cells, which selectively converted lactate into pyruvate (PA) and H_2_O_2_. The elevated H_2_O_2_ reacts with the chemiluminescent reagent CPPO, releasing energy that further activates the delivered photosensitizer Ce6 to generate highly reactive singlet oxygen. This significantly increases oxidative damage to tumor cells, induces apoptosis, and achieves the precise therapeutic effect of chemically stimulated photodynamic therapy. Li et al.^[^ ^54^ ^]^ developed a postoperative injectable thermogel loaded with BAY‐876, a widely used GLUT1‐selective competitive inhibitor, which blocks lactate excretion upstream while neutralizing lactate with sodium bicarbonate (SB). Such an approach effectively reduces the lactate content in the microenvironment, decreasing the infiltration of immune‐suppressing cells and improving the effectiveness of tumor therapy (**Figure** **3**).

Additionally, excessive accumulation of metabolites increases the endogenous metabolic burden of the tumor, driving it into a state of lethal metabolic stress, which ultimately triggers cell death. Although lactate promotes tumor development, excessive concentrations after a certain threshold can induce cellular damage. Consequently, studies have focused on the possibility of regulating tumor cell fate by exogenously providing excess lactate. Mesoporous silica nanoparticles exhibit excellent drug‐loading capacities and favorable biocompatibilities, being ideal carriers for molecules and drugs and demonstrating a wide range of applications.^[^ ^55^ ^]^ Therefore, Chavarria et al.^[^ ^56^ ^]^ synthesized and characterized LA‐loaded silica nanoparticles that effectively reduced the viability of tumor cells in vitro and significantly improved survival in a malignant glioma mouse model, showing promise as an adjuvant for in situ therapy.

Adenosine (ADO) is a common metabolite in the TME. ADO accumulation primarily occurs through a metabolic pathway in which CD73 catalyzes the conversion of AMP to ADO. Overexpression of CD73 enhances ADO production in tumor cells, immunosuppressive cells (e.g., Tregs, MDSCs), and tumor endothelial cells.^[^ ^57^ ^]^ Under tumor hypoxia, CD73 expression is upregulated, leading to excessive ADO accumulation, which promotes immune escape by activating the A2A and A2B ADO receptors in the TME. This activation inhibits effector T‐cell function and fosters the expansion of immunosuppressive cells. Consequently, targeting ADO metabolism is a promising strategy for improving tumor immunotherapy. Zhang et al.^[^ ^58^ ^]^ utilized gold–copper nanoparticles (ACS NPs) to inhibit CD73 expression and reduce ADO production. ACS NPs alleviate tumor hypoxia through the Fenton reaction, weakening ADO‐driven immunosuppression, and promoting anti‐tumor T‐cell infiltration and activity. Additionally, copper ions released from ACS NPs synergize with DSF to form cytotoxic CuET complexes, enhancing the immunotherapeutic effect of radiotherapy.

#### 4.1.4 Joint Regulation of Multilevel Metabolic Networks

Given the complexity of tumor metabolic networks, nanomedicine strategies aiming at comprehensive metabolic regulation have demonstrated significant therapeutic potential. Through multi‐target interventions and multi‐component co‐delivery, these strategies can simultaneously inhibit multiple intracellular metabolic pathways while avoiding compensatory responses. For example, after glycolysis inhibition, tumor cells may undergo lipid metabolism reprogramming and shift from glucose‐dependent metabolism to fatty acid‐dependent metabolism.^[^ ^59^, ^60^ ^]^ Based on this phenomenon, Zhang et al.^[^ ^61^ ^]^ developed a cascade‐responsive nanocapsule delivery system. This system was coupled with CPT1C siRNA (siCPT1C) via the DSF‐bonded cross‐linked anti‐VEGFR2 monoclonal antibody (aV) and surface‐loaded with the glycolysis inhibitor 2‐deoxy‐D‐glucose (2‐DG). Targeted delivery was achieved by utilizing GLUT1, which is highly expressed on the BBB and GBM cells. The system inhibited tumor cell metabolic reprogramming through the dual inhibition of the glycolysis and fatty acid oxidation (FAO) pathways, significantly reducing angiogenesis and optimizing therapeutic efficacy.

Furthermore, NADH (reduced nicotinamide adenine dinucleotide) in tumor cells can be converted to ATP as an alternative energy source to support tumor growth.^[^ ^62^ ^]^ Therefore, when glycolysis is inhibited, tumor cells compensate by increasing ATP synthesis via the mitochondrial pathway to meet physiological demands.^[^ ^63^ ^]^ Zhao et al.^[^ ^64^ ^]^ combined the PKM2 inhibitor, zeocitrin (SHK), with the aldehyde dehydrogenase 1 family member L1 (ALDH1L1) inhibitor disulfiram (DSF) and developed a hybrid albumin/lactoferrin nanosystem with BBB‐penetrating abilities (BSA/LF NP). The system simultaneously inhibited glycolysis and NADH‐ATP metabolic pathways, effectively reducing ATP production in tumor cells. Similarly, Liang et al.^[^ ^65^ ^]^ used menthol‐modified albumin as a carrier to deliver both the glycolysis inhibitor albendazole (Abz) and the mitochondrial inhibitor silver nanoparticles (SNPs) to the glioma region via BBB. The system induced tumor cell apoptosis through the combination of Abz and SNP, thus achieving comprehensive inhibition of both the glycolytic and mitochondrial pathways.

### 4.2 Neurodegenerative Diseases

#### 4.2.1 Regulation of Intracellular Metabolism

Impaired neuronal metabolic function is a fundamental contributor to cognitive decline. Restoring metabolic homeostasis in neurons, particularly through mitochondrial function and glucose metabolism regulation, is a crucial direction for current therapies.

SIRT1 plays a key role in restoring energy metabolism homeostasis by regulating mitochondrial biogenesis and OXPHOS in neurons.^[^ ^66^ ^]^ Building on this, Zhao et al.^[^ ^67^ ^]^ developed self‐assembled micelles (REn) to target the damaged BBB via receptor for advanced glycosylation end products (RAGE), enabling sequential drug delivery to the neurovascular endothelium and neurons. This approach activates the SIRT1 pathway and regulates the neuronal mitochondrial function, thereby restoring energy metabolism homeostasis. In endothelial cells, SIRT1 activation promotes NO release, improving vascular function and enhancing BBB integrity. Multimodal magnetic resonance imaging (MRI) and PET imaging revealed that REn effectively improved the cerebral blood flow (CBF) and BBB permeability, promoted glucose uptake and Aβ clearance, and ultimately enhanced cognitive function.

Li et al.^[^ ^68^ ^]^ designed a single‐atom catalyst, Pt/CeO2, wrapped in a neutrophil‐like cell membrane (HL‐60) and modified with rabies virus glycoprotein (RVG29) to enable BBB penetration and target neuroinflammatory regions. This catalyst efficiently decomposed excess ROS and targeted mitochondria through electrostatic adsorption, inducing autophagy of dysfunctional mitochondria, showing significant efficacy in treating PD. Additionally, Zhang et al.^[^ ^69^ ^]^ developed small‐sized palladium hydride (PdH) nanoparticles as hydrogen carriers and palladium‐like autocatalysts for sustained hydrogen release. These nanoparticles improved mitochondrial function and restored cellular energy metabolism by selectively scavenging highly toxic hydroxyl radicals (‐OH), significantly inhibiting neuronal apoptosis. Collectively, the therapeutic potential of nanomaterials in reestablishing metabolic homeostasis through mitochondrial function restoration was further demonstrated.

#### 4.2.2 Regulation of Cell–Cell Metabolism

Unlike other diseases, neurodegenerative disorders are characterized by significant endothelial cell dysfunction, which impairs the transport of glucose and other substances, primarily due to the downregulation of GLUT1 expression.^[^ ^70^ ^]^ This disrupted metabolic profile complicates the direct application of drug delivery strategies that rely on transporter upregulation. To address this challenge, novel delivery strategies have been proposed, employing a hypoglycemia‐induced GLUT1 feedback mechanism to modulate the endothelial transport function and enhance drug delivery efficiency.

For example, Xie et al.^[^ ^71^ ^]^ developed a glucose‐modified polymeric nanomicelle (PM) system for the treatment of AD. By inducing hypoglycemia, this system stimulated compensatory upregulation of GLUT1 expression and increased its localization in the luminal plasma membrane of brain endothelial cells. Subsequently, glucose supplementation facilitated the dynamic recycling of GLUT1 from the luminal plasma membrane to the intracellular membrane, leading to efficient delivery of the 3D6 antibody fragment (3D6‐Fab) to the brain parenchyma and effective inhibition of Aβ aggregation. Compared to the free form of 3D6‐Fab, this system enhanced drug accumulation in the brain by approximately 41‐fold. To further enhance delivery, Lei et al.^[^ ^72^ ^]^ designed a mannose‐modified PLGA‐PEG oral nanoparticle (FTY@Man NP), which achieved dual targeting of the intestinal epithelial barrier and BBB through blood glucose modulation, significantly improving both bioavailability and brain delivery efficiency. Similarly, Zhou et al.^[^ ^73^ ^]^ constructed an efficient gene delivery platform by complexing galactose‐modified polymers with siRNAs, further validating the potential of this strategy.

In addition to transporter‐based approaches, studies have highlighted the significant potential of reparative therapies targeting pericytes. By directly restoring the endothelial metabolic function, these therapies can re‐establish normal intercellular interactions, thereby maintaining neuronal function. Pericytes play a pivotal role in AD‐related vascular injury and neurodegenerative changes.^[^ ^74^ ^]^ In this context, Yang et al.^[^ ^75^ ^]^ developed a multifunctional nanomedicine, VLC@Cur‐NPs, which responds to ROS stimulation by binding the VCAM‐1 high‐affinity peptide VHS to the neuroprotective ApoE‐mimetic peptide COG1410 and curcumin (Cur) via phenylborate bonding. This design specifically targets endothelial cells, characterized by high VCAM‐1 expression and elevated ROS, enabling controlled drug release. The developed nanomedicine precisely modulated metabolic pathways in pericytes and restored the balance between glycolysis and OXPHOS, thereby enhancing endothelial‐to‐neuronal substance transport.

**Figure 4:**
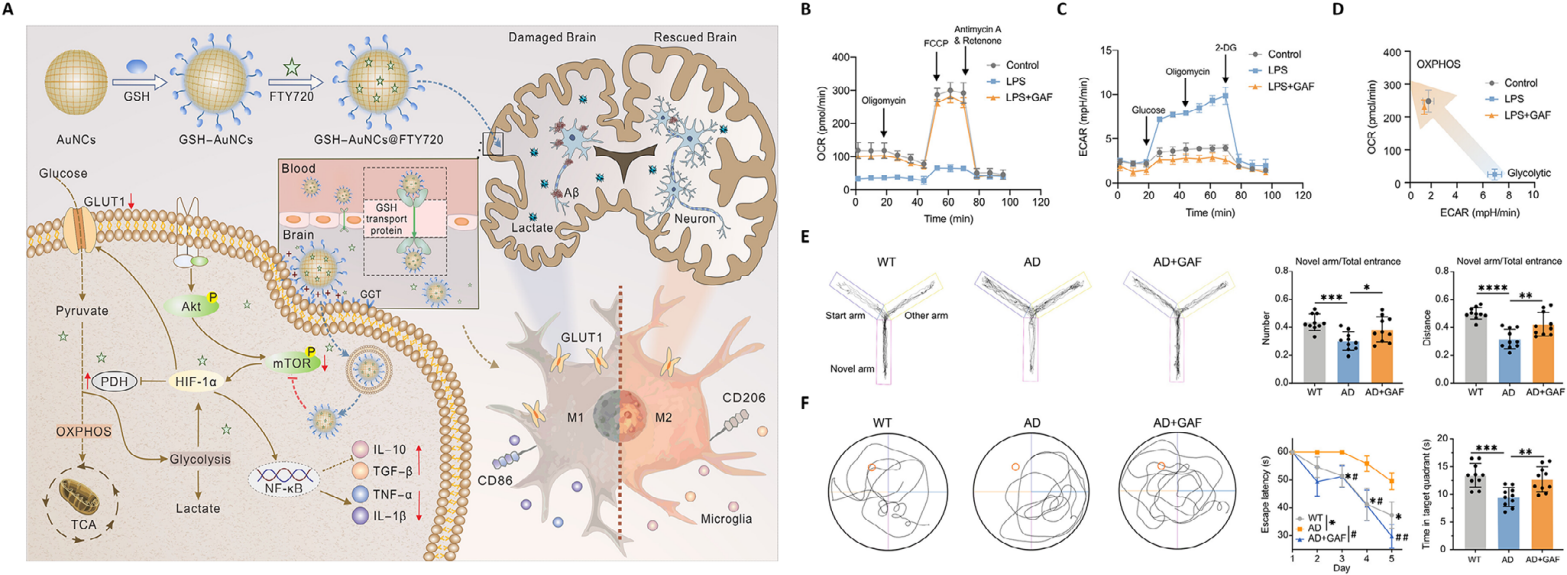
Immunometabolic reprogramming nanomodulator (GAF NPs). A) Schematic illustration of the GAF NPs and its anti‐AD mechanism. B) Representative oxygen consumption rate (OCR) profiles. C) Representative Extracellular acidification rate (ECAR) profiles. D) OCR versus ECAR analysis. E) Representative moving trajectory in the Y‐maze test and the percentage of number and distance spent in the novel arm. F) Representative swimming paths of the probe phase, escape latency during the learning phase, and time spent in the target quadrant of the probe phase in the Morris water maze test.^[^ ^81^ ^]^ Copyright 2023, American Chemical Society.

During the pathology of neurodegenerative diseases, the metabolic state of microglia exhibits highly dynamic changes,^[^ ^76^ ^]^ enabling transitions between different functional states, such as M1 pro‐inflammatory and M2 anti‐inflammatory phenotypes.^[^ ^77^ ^]^ In AD, metabolic reprogramming of microglia, driven by Aβ aggregates and ROS, induces hyperpolarization of the M1 pro‐inflammatory phenotype,^[^ ^78^ ^]^ characterized by enhanced glycolysis and impaired OXPHOS.^[^ ^79^, ^80^ ^]^ Persistent activation of microglia exacerbates chronic neuroinflammation and accelerates neuronal damage and functional degeneration by secreting inflammatory factors and neurotoxic substances. In this regard, Yang et al.^[^ ^81^ ^]^ developed glutathione‐functionalized gold nanocages (Au NCs) as mTOR‐targeted nanopreparations (GAF NPs), combined with fingolimod hydrochloride (FTY720), to modulate microglial metabolism. By reversing the shift from glycolysis to OXPHOS and blocking the Akt/mTOR/HIF‐1α signaling pathway, these nanoparticles effectively improved glucose utilization in the brain, inhibited inflammatory response, and enhanced cognitive function in AD mice (**Figure** **4**). Such findings highlight the efficiency of metabolic intervention in correcting microglial dysfunction.

#### 4.2.3 Regulation of Cell–Microenvironment Metabolism

Based on the interactions between microglia and the brain microenvironment during AD progression, Lu et al.^[^ ^82^ ^]^ developed a ROS‐responsive polymeric micelle system (APLB/CUR), which effectively restores the homeostasis of the oxidative and inflammatory microenvironment, achieving functional remodeling of microglia in the early stages of AD. By mimicking the Aβ transport pathway, the micelles target the diseased region and achieve a synergistic effect through polymer‐based ROS scavenging and Aβ inhibition, providing multilevel interventions against microenvironmental stimuli. This multi‐targeting strategy significantly modulated the brain microenvironment, offering neuroprotection and functional reprogramming of microglia, leading to a marked reduction in Aβ plaque load and improvement in cognitive function. This study highlights microglia as a promising key target for early AD treatment, with microenvironmental modulation holding the potential to precisely regulate their functional state and intervene in the disease process.

#### 4.2.4 Joint Regulation of Multilevel Metabolic Networks

Within the framework of intricate metabolic interactions across multiple cell types, multi‐targeted nano‐delivery systems exhibit considerable advantages. By precisely orchestrating the metabolic functions and intercellular signaling pathways, these systems can effectively restore metabolic equilibrium. Zhang et al.^[^ ^83^ ^]^ pioneered an innovative nanodrug that successfully traverses the BBB using an MMP9‐activated cell‐penetrating peptide (mAP). This system selectively and simultaneously targets cerebral vascular endothelial cells, neurons, and microglia, leveraging magnesium ions (Mg^2^⁺), a naturally occurring calcium antagonist, in conjunction with cyclophilin D (CypD) siRNA, a key regulator of the mitochondrial permeability transition pore (mPTP). This approach corrects mitochondrial dysfunction across these three cell populations, restores their metabolic homeostasis, and attenuates the neuropathological alterations associated with neurodegenerative diseases.

**Figure 5:**
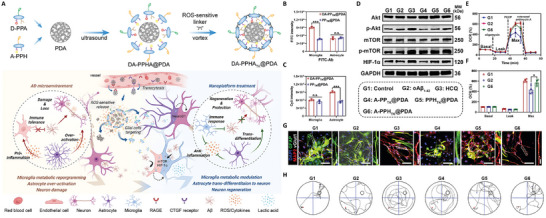
A multi‐targeted peptide‐drug conjugate‐based nanoplatforms. A) Self‐assembly process of nanoplatform and the mechanism of glial cells‐targeting therapy of nanoplatform in advanced stage AD. B,C) Examination of targeting ability of B) FITC labeled A‐PPH and C) Cy5 labeled D‐PPA in primary microglia and astrocyte. D) Changes in Akt/mTOR/HIF‐1α pathway expression in microglia with different treatments. E) Oxygen consumption profile of microglia in different treatments. F) Quantification of basal respiration, proton leakage and maximal respiration in microglia of different treatments. G) Representative confocal microscopic images of expression of mature neuronal markers and functional transporters in different treated primary astrocytes. H) Representative swimming paths of mice in Morris water maze after three months of different treatments.^[^ ^84^ ^]^ Copyright2024, Wiley‐VCH.

Astrocytes play a crucial role in regulating energy metabolism within the central nervous system and exhibit dual metabolic characteristics in AD. On one hand, disruptions in lactate secretion and glutamine metabolism by astrocytes hinder the energy supply to neurons. On the other hand, astrocytes possess a certain degree of metabolic plasticity, offering a potential target for metabolic intervention. In this context, Liu et al.^[^ ^84^ ^]^ developed a modular nanomedicine system that targets both astrocytes and microglia for comprehensive metabolic regulation. Hydroxychloroquine (HCQ) and all‐trans retinoic acid (ATRA) were selected as model agents for immune activation and cell differentiation induction, respectively. Covalently linked to polymeric backbones (D‐PPA and A‐PPH) with a dopamine (PDA) core, these agents were released in the oxidative stress microenvironment through degradation of the nanostructures. HCQ was delivered to microglia to enhance their immune response and improve mitochondrial metabolism, while ATRA selectively targeted astrocytes to induce their transdifferentiation into functional neurons, thereby alleviating inflammation. The system efficiently restored astrocytic energy metabolism, improved lactate‐glucose coupling, and reduced glutamate toxicity, which in turn supported neuronal energy supply and facilitated recovery of the neurological function (**Figure** **5**).

### 4.3 Cerebrovascular Diseases

#### 4.3.1 Regulation of Intracellular Metabolism

The pathological progression of cerebrovascular diseases, such as ischemic stroke, profoundly disrupts the cellular metabolic function, as evidenced by the impaired glucose metabolism, mitochondrial dysfunction, and imbalances between energy supply and demand. Specific metabolic dysregulation in key cells directly contributes to disease progression and exacerbates neurological decline. Consequently, restoring the metabolic function of targeted cells has become a critical therapeutic strategy.

Mitochondria play a pivotal role in ischemic stroke. At the onset of the condition, insufficient blood and oxygen supply impairs mitochondrial function, triggering oxidative stress. Excessive ROS can damage cellular structures,^[^ ^85^ ^]^ leading to neuronal injury and increasing the risk of neuronal apoptosis.^[^ ^86^ ^]^ Moreover, mitochondrial damage is further exacerbated by ischemia‐reperfusion injury (CIRI) following pharmacological interventions, such as tissue plasminogen activator (tPA)‐induced CIRI.^[^ ^85^ ^]^

**Figure 6:**
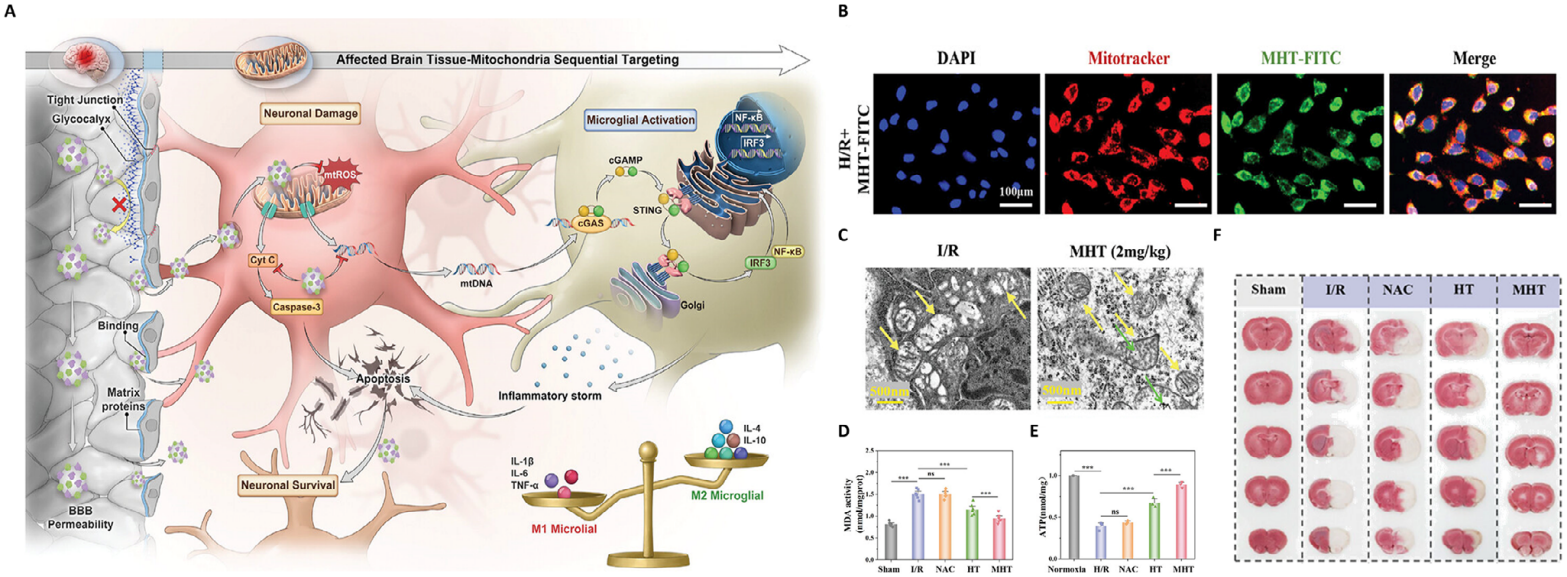
Sequential targeting nanomedicine (MHT) of neuronal mitochondria. A) Schematic mechanism and characteristics of MHT. B) Examination of the mitochondrial targeting ability of MHT‐FITC. C) Transmission electron microscopy (TEM) images of neuronal mitochondria (indicated in yellow) in the ischemia/reperfusion (I/R) and MHT treatment groups. D) Triphenyltetrazolium chloride (TTC) staining of brain tissue. E) Quantification of malondialdehyde levels in brain tissue, an indicator of oxidative damage. F) ATP production of cells under different treatments.^[^ ^87^ ^]^ Copyright 2024, Wiley‐VCH.

Consequently, mitochondrial function restoration has become a primary focus in neuroprotection research. Wang et al.^[^ ^87^ ^]^ developed tannic acid (TA)‐melanin‐modified nanoparticles (MHT), an innovative mitochondria‐targeting nanomedicine. TA enables sequential targeting of damaged brain tissue and mitochondrial outer membrane proteins via its specific binding to BBB, while melanin enhances the antioxidant capacity of the system. MHT significantly reduced neuronal apoptosis by targeting mitochondria to scavenge reactive ROS, repair mitochondrial function, and regulate the cGAS‐STING pathway to inhibit oxidative stress and immune‐inflammatory responses induced by CIRI. Furthermore, MHT retained significant efficacy and biosafety even at ultra‐low doses (**Figure** **6**). To further enhance BBB penetration, some studies have employed nasal administration to bypass the BBB and improve delivery efficiency. Specifically, mitochondria‐targeting peptide Bendavia (SS‐31)‐modified nanocarriers (SPNPs) were developed and encapsulated in a temperature‐sensitive hydrogel for intranasal delivery.^[^ ^88^ ^]^ In vivo, the thioredoxin‐crosslinked skeleton of SPNPs ruptured in response to high ROS concentrations in the focal area, releasing geranylgeranyl and alleviating mitochondrial dysfunction, thereby demonstrating promising therapeutic potential.

The recent development of functional nanomaterials has opened new avenues for the precise treatment of brain diseases, particularly in overcoming the limitations of conventional therapies.^[^ ^89^ ^]^ Nanomaterials with enzyme‐mimicking activities, such as manganese dioxide nanoenzymes and metal‐organic framework (MOF) nanoenzymes, have been extensively studied, demonstrating significant potential in combating oxidative stress and enhancing cellular functions.^[^ ^90^ ^]^ Cerium nanoenzymes (CeNZs) have emerged as highly effective agents for ROS modulation, owing to their reversible Ce^3+^/Ce^4+^ valence switching properties. CeNZs efficiently scavenge superoxide anion (‐O^2−^), hydrogen peroxide (H_2_O_2_), and hydroxyl radicals (‐OH) by mimicking the functions of superoxide dismutase (SOD) and catalase (CAT). With superior efficiency and reproducibility compared to traditional scavengers, CeNZs have found widespread application in ROS regulation and the treatment of related diseases.^[^ ^91^ ^]^ Liao et al.^[^ ^92^ ^]^ developed a mitochondria‐targeted nano‐delivery system using DSPE‐encapsulated CeNZs to enhance the blood circulation time and bioavailability. Further modified with TPP, mitochondria‐targeted CeNZs served as drug carriers to deliver roflumilast, a PDE4 inhibitor, thereby providing enhanced neuroprotection.

Moreover, Quan et al.^[^ ^93^ ^]^ enhanced ATP and NADPH production through a neutrophil‐mimicking, microalgae‐derived upconverting photosynthetic nanosystem designed to aid neurons in restoring their energy metabolism. The system comprised upconverted nanoparticles encapsulated in vesicle‐like membranes, further functionalized with ROS‐responsive thrombolytic tPA to ensure high target specificity. Upon exposure to ROS at the thrombus site, the nanosystem rapidly exhibited thrombolytic activity through surface‐modified tPA and activated ATP and NADPH production via near‐infrared II (NIR‐II) laser stimulation. This process inhibited inflammatory cell infiltration, platelet activation, oxidative stress, and neuronal damage, underscoring the superior efficacy of nanocarriers when combined with other therapeutic strategies.

#### 4.3.2 Regulation of Cell–Cell Metabolism

In the early phase of stroke, the interruption of cerebral blood flow triggers an energy crisis, to which brain endothelial cells respond by transiently upregulating glucose transporter proteins to enhance glucose uptake. Building on this, Liu et al.^[^ ^94^ ^]^ developed a “two‐for‐one” nano‐delivery system using the neuroprotective polyphenol myricetin (Myr) as a backbone. Co‐assembled with polyvinylpyrrolidone (PVP), the system forms needle‐like nanostructures (PM NPs) and incorporates Ce ions for enhanced stability. The resulting colloidal nanoparticles (PC NPs) facilitated drug delivery across the BBB by interacting with the overexpressed glucose transporter proteins under hypoxic conditions, thereby supplying the stroke‐affected region with essential energy.

#### 4.3.3 Regulation of Cell–Microenvironment Metabolism

During the progression of ischemic stroke, hypoxia is a predominant pathological feature of the microenvironment. Consequently, numerous nanomedicines have been developed to alleviate oxygen deficiency in ischemic focal areas, such as polyfluorocarbon nanocarriers, which directly mitigate the hypoxic microenvironment by carrying oxygen.^[^ ^95^ ^]^ He et al.^[^ ^96^ ^]^ developed a drug‐free mimetic nanocarrier (MFP), consisting of polyfluorocarbon combined with Pluronic P123, which was camouflaged by M2‐type microglia membranes. This formulation alleviated metabolic stress on endothelial cells by enhancing oxygen supply to ischemic regions while modulating glucose‐oxygen metabolic pathways. Furthermore, Pluronic P123 inhibited MMP‐9 secretion, reduced structural damage to the BBB, restored metabolic homeostasis in the stroke‐affected region, and ultimately reduced infarct size, thus improving neurological deficits.

Disturbances in amino acid metabolism, particularly in the glutamate and glutamine cycles, play a crucial role in the pathology of ischemic stroke. Hypoxia and oxidative stress lead to neuronal energy depletion and ion pump dysfunction, triggering glutamate release and activation of N‐methyl‐D‐aspartate receptors (NMDAR), leading to excitotoxic responses and exacerbating neurological damage. Given that the glutamine cycle regulates NMDAR activation and prevents neuronal damage by removing the metabolite NH_4_^+^, its disruption leads to NH_4_^+^ accumulation in the microenvironment, further activating NMDAR and creating a vicious cycle. Meanwhile, the activated NMDAR promotes Fe^2+^ uptake and accumulation in neurons,^[^ ^97^, ^98^ ^]^ ultimately leading to neuronal death. To address these issues, Wang et al.^[^ ^99^ ^]^ combined Pt nanoclusters (Pt NCs) and transferrin (Apo‐LF) to develop a biomimetic nanosystem, Pt@LF. In a rat model of transient cerebral ischemia (tMCAO), Pt@LF effectively penetrated ischemic regions, scavenged ROS, chelated iron, and removed excess NH_4_^+^ from the microenvironment, thereby inhibiting iron accumulation and NMDAR overactivation and ameliorating the inflammatory microenvironment. Such results highlight the potential of Pt@LF as a promising clinical strategy.

In addition to therapeutic strategies, novel diagnostic methods have been developed to monitor metabolic alterations in stroke. Acute metabolic collapse can trigger abnormal consumption and accumulation of substances. In the pathological state of stroke, abnormal cysteine metabolism is a hallmark of metabolic dysfunction and is closely linked to oxidative stress, neuroinflammatory responses, and BBB disruption. Stroke‐induced hypoxia and the ensuing energy crisis in brain tissue disrupt the cysteine metabolic pathways, thereby affecting nervous system function. Based on this metabolic alteration, Wang et al.^[^ ^100^ ^]^ designed a novel fluorescent nanoprobe (S‐DCM‐NIR(835)) employing an intramolecular charge transfer (ICT) mechanism and Knoevenagel condensation. Through a Michael addition reaction, the S‐DCM‐NIR(835) probe specifically recognizes cysteine and accurately reflects concentration changes in the stroke region due to metabolic disturbances. The developed probe enables real‐time monitoring of metabolic changes in the stroke lesion area and assessment of stroke severity via response time, as well as quantifies dynamic cysteine concentration changes, providing an accurate metabolic marker for early stroke diagnosis and pathological assessment.

#### 4.3.4 Joint Regulation of Multilevel Metabolic Networks

**Figure 7:**
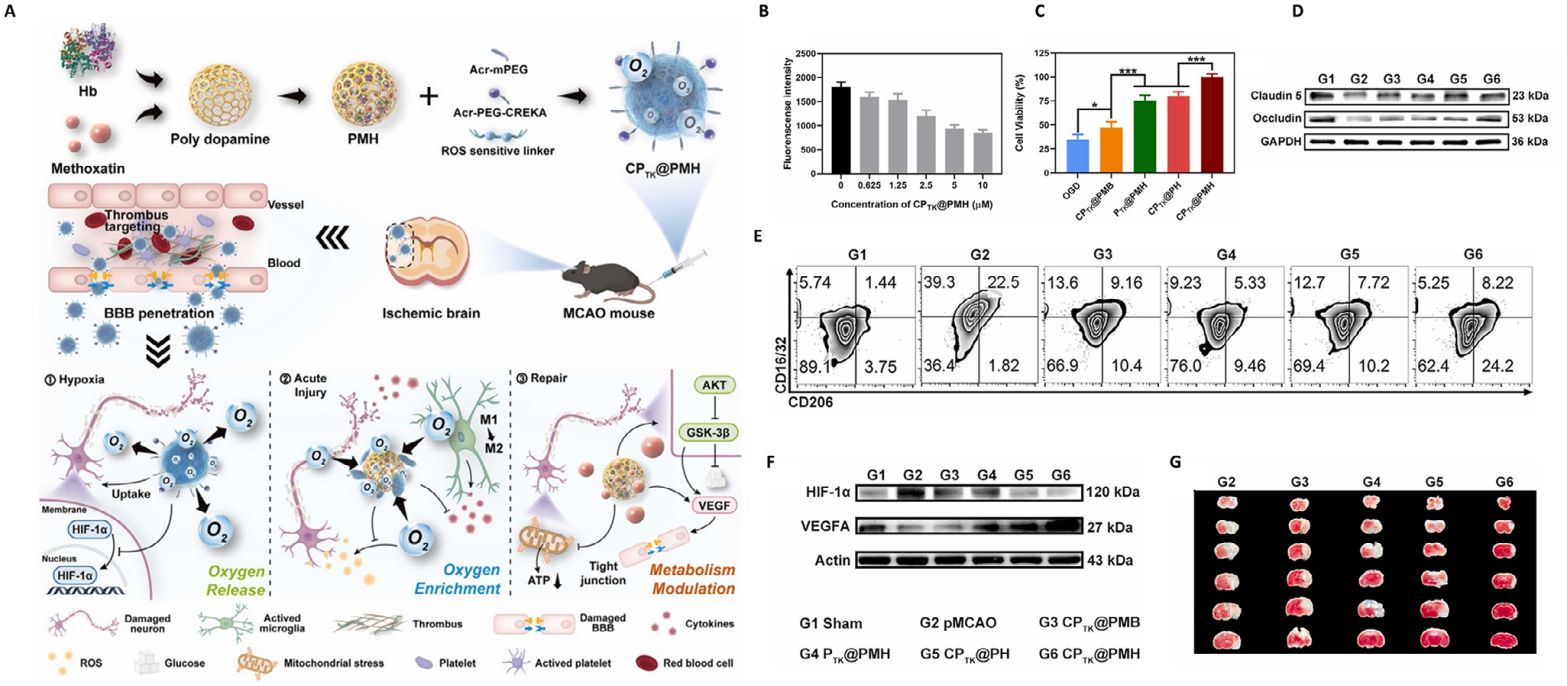
Bioinspired nanoerythrocytes (CPTK@PMH) for metabolic microenvironment remodeling. A) Schematic diagram of CPTK@PMH nano‐microenvironmental regulation of erythropoiesis and ischemic brain metabolism. B) Oxygen probe to investigate the oxygen production capacity of CPTK@PMH in SH‐SY5Y cells. C) Examine the changes in cell viability of SH‐SY5Y cells after different treatments. D) Examination of the change in phenotypic polarization state of microglia after CPTK@PMH treatment. E) Western blotting to examine the effect of different treatments on the expression of tight junction proteins in bEnd.3 cells. F) Western blotting for the expression of hypoxia‐related pathways in the brains of pMCAO model mice. G) Representative TTC staining images of brain sections.^[^ ^101^ ^]^ Copyright 2023, Elsevier Ltd.

By strategically designing responsive structures for nanodrugs, intelligent drug release can be achieved while simultaneously modulating multiple features of the metabolic microenvironment. This approach provides a novel avenue for the comprehensive regulation of the metabolic network. Liu et al.^[^ ^101^ ^]^ developed a biomimetic nano‐erythrocyte system (CPTK@PMH) with dual microthrombus‐targeting and metabolic microenvironment remodeling capabilities. The system integrates hemoglobin and pyrroloquinoline quinone (PQQ) within dopamine nanocages to form a polymer layer, enhancing stability. Following microthrombus‐targeting peptide modification, CPTK@PMH selectively accumulates in the ischemic core, where it releases oxygen upon saturation, alleviating the hypoxic microenvironment. During reperfusion, nano‐erythrocytes inhibit ROS generation by integrating excess oxygen, mitigating acute injury caused by the pro‐inflammatory environment. Furthermore, PQQ‐responsive release activates the Akt/GSK‐3β pathway, remodeling neuronal glucose metabolism, restoring energy supply, and enhancing neuroprotection (**Figure** **7**).

## 5 Conclusion and Perspectives

Although the pathological mechanisms and metabolic characteristics of brain diseases vary significantly, the pathological processes induced by multi‐level metabolic dysregulation share common features. Specifically, the disruption of core cellular metabolic functions leads to disturbances in intercellular interactions, which ultimately foster a malignant microenvironment conducive to disease progression. This process involves not only the transport, utilization, and excretion of substances but also incorporates complex mechanisms such as cellular signal transduction, intercellular communication, and dynamic regulation of the microenvironment. Given that the brain is the organ with the highest metabolic demand, a deeper understanding of the metabolic mechanisms underlying these diseases is essential for advancing therapeutic strategies.

In light of these factors, functionalized nano‐delivery systems demonstrate significant advantages in multi‐target integrated regulation, co‐delivery of multiple components, and multi‐stage responsive modulation.^[^ ^102^ ^]^ The strategy of “joint regulation of multilevel metabolic networks” enables simultaneous intervention across multiple metabolic pathways, coordinating regulation of heterogeneous cell populations and pathological microenvironments. This approach aligns well with the complex and dynamic progression of brain diseases, showing significant application potential and providing promising avenues for the development of diagnostic and therapeutic solutions for brain diseases.

Although nano‐delivery systems have shown significant promise in the field of metabolic regulation, three primary challenges remain:

1. Achieving precise control over nanocarrier development remains a critical challenge. Given the essential role of metabolism in cellular function, it is crucial to avoid unintended interference with non‐target cells and minimize the risk of side effects and toxicity. Therefore, there is an urgent need for comprehensive studies on the metabolic differences between cells and their underlying mechanisms, providing essential insights for the precise design and optimization of nano‐delivery systems.
2. Most existing nano‐delivery strategies predominantly target single metabolic pathways, while comprehensive approaches that integrate regulation across intracellular, intercellular, and microenvironmental metabolic networks remain underdeveloped. Current multilayer metabolic regulation strategies predominantly focus on the “energy metabolism” level, such as glycolysis and lipid oxidation. Many approaches rely on empirical, parallel stacking of interventions, lacking a precise framework grounded in the pathological network topology. Consequently, they lack adaptability to the dynamic metabolic reprogramming throughout disease progression. Moreover, the effectiveness of nano‐delivery systems in conjunction with other therapeutic strategies remains underexplored. The strategies outlined in the previous section indicate that metabolic modulation‐based approaches are in the early stages for certain disease areas, particularly functional and episodic disorders. Encouragingly, the metabolic mechanisms of certain diseases are gradually being uncovered. For instance, in epilepsy treatment, strategies targeting glucose or lactate metabolism in the brain hold promise for significantly reducing seizure frequency and enhancing neurological recovery.^[^ ^103^, ^104^ ^]^ However, the metabolic mechanisms underlying brain diseases, such as amyotrophic lateral sclerosis (ALS), have yet to be fully elucidated due to the limited patient population and the complexity of the pathological processes.^[^ ^105^ ^]^ Nevertheless, the development of nanoplatforms remains highly significant, offering both a technological foundation and a feasible strategic framework for future investigation and intervention in metabolic mechanisms.
3. “Translatability” remains a critical bottleneck in advancing metabolism‐targeted nanomedicine strategies toward clinical application.^[^ ^106^ ^]^ Although several nanotherapeutics have demonstrated preliminary safety and BBB‐penetrating capabilities in early‐phase clinical trials, brain metabolism‐focused nanomedicine remains in its infancy, with translational attempts being limited. Notably, BXQ‐350, a nanovesicle formulation targeting sphingolipid metabolism, has shown promise by prolonging progression‐free survival in patients with recurrent glioma (NCT01906385), thereby providing initial evidence of clinical feasibility for metabolism‐based interventions. Metabolic pathways, being relatively conserved and quantifiable targets, may improve patient stratification and treatment consistency, thereby enhancing translational efficiency. However, their dynamic and adaptive nature complicates precise intervention, especially in the absence of real‐time metabolic imaging and reliable biomarkers. Furthermore, the complexity and cost of nanoparticle production and quality control remain significant barriers. Future development must therefore balance biological efficacy with manufacturability. Standardized, scalable delivery systems integrated with clinical pharmacological management will be essential to advance these promising strategies toward clinical application.

Looking ahead, intelligent nano‐drug delivery systems featuring multi‐target, multi‐component, and multi‐process regulation, coupled with various combination strategies, are expected to achieve breakthroughs in both precision and therapeutic efficiency for brain diseases. Such advances will drive in‐depth innovation of personalized therapeutic solutions and clinical applications (Supporting Information).

## Conflict of Interest

The authors declare no conflict of interest.

## Supporting information



Supporting Information
