# Supplementary material for: Targeting Multilayered Metabolic Networks in Brain Diseases: Emerging Perspectives on Nanodelivery Strategies
Source: Adv Sci (Weinh). 2025 Sep 25;12(41):e03645. doi: 10.1002/advs.202503645 (PMC12591122; doi:10.1002/advs.202503645)
Supplement: Supplementary file 1 — Supporting Information [file ADVS-12-e03645-s001.docx]

**Supporting information**

**Contents:**

**Table S1.** Glossary of Abbreviations

**Table S1. Glossary of Abbreviations**

| **Abbreviation** | **Full Name** |
| --- | --- |
| 18F-FDG | Fluorodeoxyglucose labeled with fluorine-18  (used in PET imaging to monitor glucose metabolism) |
| 2-DG | 2-deoxy-D-glucose |
| 3BP | 3-Bromopyruvate |
| A2A / A2B | Adenosine A2A/A2B Receptor |
| AD | Alzheimer’s disease |
| ADO | Adenosine |
| ALDH1L1 | Aldehyde Dehydrogenase 1 Family Member L1 |
| AMP | Adenosine monophosphate |
| Ang | Angiotensin-converting enzyme-2 |
| ApoE | Apolipoprotein E |
| ATP | Adenosine triphosphate |
| ATRA | All-trans retinoic acid |
| BBB | Blood-brain barrier |
| bEnd.3 | Brain Endothelial Cell Line |
| BMDM | Bone marrow-derived macrophages |
| CBF | Cerebral Blood Flow |
| CD | Cyclodextrin |
| Ce6 | Chlorin e6 |
| CIRI | Cerebral Ischemia-Reperfusion Injury |
| CPT1C | Carnitine palmitoyltransferase 1C |
| DAPI | 4',6-diamidino-2-phenylindole |
| DHCR7 | 7-Dehydrocholesterol reductase |
| DSF | Disulfiram |
| EB | Exosomes derived from brain metastatic breast cancer |
| ECAR | Extracellular acidification rate |
| FAO | Fatty Acid Oxidation |
| FCM | Flow cytometry |
| GAPDH | Glyceraldehyde-3-phosphate dehydrogenase |
| GBM | Glioblastoma |
| GGT | γ-Glutamyl transpeptidase |
| GLUT1 | Glucose transporter 1 |
| GLUT3 | Glucose transporter 3 |
| GOx | Glucose oxidase |
| GRMs | Glioma-associated microglia/macrophages |
| GSH | Glutathione |
| GSM | Glioma-supportive macrophages |
| H₂O₂ | Hydrogen peroxide |
| HCQ | Hydroxychloroquine |
| HK-II | HK-II |
| HL-60 | Human promyelocytic leukemia cell line |
| I/R | Ischemia/Reperfusion |
| ICT | Intramolecular Charge Transfer |
| IFN-γ | Interferon gamma |
| IS | Ischemic stroke |
| ITM | Immunosuppressive tumor microenvironment |
| Ki67 | Marker of proliferation |
| LA | Lactic acid |
| LAT1 | Large amino acid transporter 1 |
| LF | Lactoferrin |
| LND | Lonidamine |
| LOX | Lactate oxidase |
| MCT1 | Monocarboxylate transporter 1 |
| MDA | Malondialdehyde |
| MMP-9 | Matrix Metalloproteinase-9 |
| MOF | Metal-Organic Framework |
| MRI | Magnetic Resonance Imaging |
| NADH | Nicotinamide adenine dinucleotide (reduced form) |
| NADPH | Nicotinamide adenine dinucleotide phosphate (reduced form) |
| NC | Nanocapsule |
| NIR-II | Near-Infrared II |
| NMDA | N-methyl-D-aspartate (receptor) |
| NMDAR | N-methyl-D-aspartate Receptor |
| NO | Nitric oxide |
| NRP-1 | Neuropilin-1 |
| OCR | Oxygen Consumption Rate |
| OXPHOS | Oxidative Phosphorylation |
| PD | Parkinson’s disease |
| PDA | Polydopamine |
| PDE4 | Phosphodiesterase 4 |
| PD-L1 | Programmed death-ligand 1 |
| PET | Positron emission tomography |
| PKM2 | Pyruvate kinase M2 |
| RAGE | Receptor for Advanced Glycation End Products |
| ROS | Reactive oxygen species |
| RVG29 | Rabies virus glycoprotein 29 |
| SB | Sodium bicarbonate |
| SH-SY5Y | Human neuroblastoma cell line |
| siRNA | Small interfering RNA |
| SIRT1 | Sirtuin 1 |
| SOD | Superoxide Dismutase |
| TA | Tannic Acid |
| TAMs | Tumor-associated macrophages |
| TEM | Transmission Electron Microscopy |
| TLR7/8 | Toll-like receptor 7 and 8 |
| tMCAO | Transient Middle Cerebral Artery Occlusion |
| TME | Tumor microenvironment |
| TMZ | Temozolomide |
| TNF-α | Tumor necrosis factor alpha |
| TPP | Triphenylphosphine |
| Tregs | Regulatory T cells |
| TTC | 2,3,5-Triphenyltetrazolium chloride |
| TUNEL | Terminal deoxynucleotidyl transferase dUTP nick end labeling |
| VCAM-1 | Vascular Cell Adhesion Molecule-1 |
| VEGFR2 | Vascular endothelial growth factor receptor 2 |
| ZO-1 | Zonula occludens-1 |
